# Evaluation of whole-body MRI with diffusion-weighted sequences in the staging of pediatric cancer patients

**DOI:** 10.1371/journal.pone.0238166

**Published:** 2020-08-27

**Authors:** Alex Dias de Oliveira, Guilherme Heidi Yto de Souza, Camila Pinto Brito de Figueiredo Guimarães, Marcos Duarte Guimarães, Cecília Maria Lima da Costa, Fábio Henrique de Gobbi Porto, Rubens Chojniak

**Affiliations:** 1 Imaging Department, A.C. Camargo Cancer Center, São Paulo, SP, Brazil; 2 Pediatric Department, A.C. Camargo Cancer Center, São Paulo, SP, Brazil; 3 Neurology Department, University of São Paulo, São Paulo, SP, Brazil; Northwestern University Feinberg School of Medicine, UNITED STATES

## Abstract

**Background:**

The purpose of this study was to determine whether whole-body MRI (WBMRI) with diffusion-weighted sequences, which is free of ionizing radiation, can perform as well as traditional methods when used alone for staging or follow-up of pediatric cancer patients.

**Methods:**

After obtaining approval from our institutional research ethics committee and appropriate informed consent, we performed 34 examinations in 32 pediatric patients. The examinations were anonymized and analyzed by two radiologists with at least 10 years’ experience.

**Results:**

The sensitivity and specificity findings, respectively, were as follows: 100% and 100% for primary tumor; 100% and 86% for bone metastasis; 33% and 100% for lung metastasis; 85% and 100% for lymph node metastasis; and 100% and 62% for global investigation of primary or secondary neoplasias. We observed excellent interobserver agreement for WBMRI and excellent agreement with standard staging examination results.

**Conclusions:**

Our results suggest that pediatric patients can be safely imaged with WBMRI, although not as the only tool but in association with low-dose chest CT (for subcentimeter pulmonary nodules). However, additional exams with ionizing radiation may be necessary for patients who tested positive to correctly quantify and locate the lesions.

## Introduction

Most of the imaging methods used in the initial evaluation and follow-up of pediatric cancer patients emit ionizing radiation, which has been shown to cause DNA damage and, consequently, increases the risk of carcinogenesis. In addition, its effects are cumulative, and must be used judiciously in pediatric patients due to their longer life expectancy [1–5].

The capacity of magnetic resonance imaging, which is free of ionizing radiation, to differentiate between benign and malignant tumors, to help in staging and to assess therapeutic response in cancer patients has been described in a number of studies [6–12].

Oncological studies of whole-body magnetic resonance imaging (WBMRI) have demonstrated that it is superior to bone scintigraphy for detecting bone metastases and that its performance is comparable to metaiodobenzylguanidine (MIBG) scintigraphy in detecting extra-osseous metastases of neuroblastoma [3, 6, 13, 14]. In 2012, Marylin et al. published a multicenter prospective study comparing the accuracy of WBMRI vs. standard staging in pediatric oncology patients, concluding that the noninferior accuracy for diagnosis of distant metastasis in patients with common pediatric tumors was not established for the use of whole-body MR imaging compared with conventional methods [15]. An important limitation of this study was that diffusion sequences were not used since they were not available for whole body imaging when the study began in 2006. In an effort to reduce up to 70% of the radiation dose, studies have compared the fusion of PET images with WBMRI (PET-MRI) vs. PET-CT. Their results indicated that this modality could become a future reference in pediatric cancer patients [16–18].

Other studies have established comparisons similar to ours, between WBMRI versus PET-CT or bone scintigraphy, but in the setting of specific types of cancer or clinical situations (for example, detection of bone metastasis) [23–27]. We resume this discussion, correlating to our findings later in this manuscript.

We believe that WBMRI with diffusion can serve as an alternative tool that avoids the risks of ionizing radiation in the staging and post-therapeutic follow-up of children and adolescents, with results comparable to currently used imaging tests. Therefore, the purpose of this study was to evaluate whether the performance of WBMRI with diffusion is comparable to traditional methods in the staging and follow-up of pediatric patients.

## Materials and methods

This cross-sectional, prospective, single-centered, non-randomized study was conducted between February 2013 and July 2014 at the A. C. Camargo Cancer Center (São Paulo, Brazil) with pediatric oncology patients who, after obtaining approval from our institutional research ethics committee and appropriate informed consent, underwent imaging examinations for staging or follow-up. The inclusion criteria were: 1) a confirmed histological diagnosis of cancer in patients up to 18 years of age; 2) scheduled imaging tests for cancer staging or follow-up; 3) written informed consent by parents or guardians. The exclusion criteria were: 1) contraindications to MRI; 2) pregnancy or lactation; 3) lack of cooperation regarding the requested maneuvers or patients who could not maintain a decubitus position; 4) the need for sedation to perform the exam, except when requested by the attending physician.

Eligible patients were recruited to undergo WBMRI with the T1, T2 STIR and diffusion sequences in a 1.5T system (Signa Excite HD; GE Healthcare, Milwaukee, USA) with a body coil and maximal gradient power of 33 mT/m and a pulse rate of 160 mT/m/s.

Oncologic staging and follow-up examinations (reference standard) included PET-CT (in a 16-channel GE Discovery 600 scanner after intravenous administration of 0.12 mCi of ^18^F-FDG per kg) bone scintigraphy with ^99^mTc-MDP, and CT with nonionic venous contrast (in a 16-channel Philips Brilliance Big Bore scanner).

Further detail on imaging exams protocol can be found in S1 Appendix.

The interval between the reference exams and WBMRI was less than 30 days.

### Image analysis

The images were analyzed by two examiners with more than ten years’ experience in oncological radiology, who only obtained information about neoplasm type during staging or follow-up. Each examiner performed the evaluation independently and was asked to record the number, size and location of the lesions. When divergent findings were reported by the observers, a joint assessment of the images was performed to reach a consensual opinion.

### Statistical analysis

The image exams involved in diagnostic or follow-up protocols (PET-CT, CT and scintigraphy), associated with clinical evaluation and occasional invasive procedures (bone marrow biopsy), were considered the reference standard, and their results were compared to those of WBMRI with diffusion.

Categorical variables were described as frequencies and percentages, while quantitative variables were described as measures of central tendency (mean and median) and dispersion (standard deviation and interquartile range).

To evaluate the agreement between WBMRI and clinical-radiological staging exams, we used the Kappa statistic (K). A K-value of 0 as indicating no agreement and 0–0.20 as slight, 0.21–0.40 as fair, 0.41–0.60 as moderate, 0.61–0.80 as substantial, and 0.81–1 as almost perfect agreement 28. All statistical analyses were performed with SPSS version 17.

## Results and discussion

We performed 34 WBMRI exams in 32 patients (two patients underwent two exams). The mean patient age was 13.3 years (range: 3–18) and 50% were male. A total of 14 malignant tumors were found in the sample, with osteosarcoma being the most frequent (Table 1). Table 2 shows the distribution of cases in order of entry into the study, the diagnoses and the reference exams.

**Table 1:** Frequency of neoplasms in the study.

Neoplasia	Frequency
Osteosarcoma	12 (35.2%)
Lymphoma	7 (20.5%)
Rhabdomyosarcoma	3 (8.8%)
Li-Fraumeni and multiple tumors	2 (5.8%)
Ovarian tumor	2 (5.8%)
Peritoneal Pseudomyxoma	1 (2.9%)
Wilms’ Tumor	1 (2.9%)
Chondroblastoma	1 (2.9%)
Retinoblastoma	1 (2.9%)
Ewing sarcoma	1 (2.9%)
Neuroblastoma	1 (2.9%)
Endodermal sinus tumors	1 (2.9%)
PNET	1 (2.9%)

**Table 2:** The distribution of cases, diagnoses and exams performed in standard staging.

Case	Diagnostic	Exams performed	Age (years)/Sex
1	Embryonal Rhabdomyosarcoma	PET-CT/Bone scintigraphy/Thorax CT	3 / F
2	Osteosarcoma	PET-CT/Bone scintigraphy/Thorax CT	11 / M
3	Osteosarcoma	Bone scintigraphy/Thorax CT	7 / F
4	Endodermal sinus tumors	Bone scintigraphy/Thorax, abdominal and pelvic CT	3 / F
5	Hodgkin’s Lymphoma	PET-CT/Neck, thorax, abdominal and pelvic CT	5 / F
6	Osteosarcoma	Bone scintigraphy/Thorax CT	13 / M
7	Osteosarcoma	PET-CT/Bone scintigraphy/Thorax CT	18 / M
8	Osteosarcoma	PET-CT/Thorax CT	6 / M
9	Osteosarcoma	PET-CT/Bone scintigraphy/Thorax CT	11 / F
10	Osteosarcoma	Bone scintigraphy/Thorax CT	11 / F
11	Osteosarcoma	Bone scintigraphy/Thorax CT	18 / M
12	Hodgkin’s Lymphoma	PET-CT	12 / M
13	Osteosarcoma	PET-CT/Bone scintigraphy/Thorax CT	18 / M
14	Li-Fraumeni and multiple tumors	Bone scintigraphy/Thorax CT	18 / F
15	Hodgkin’s Lymphoma	PET-CT/ Thorax, abdominal and pelvic CT	14 / F
16	Osteosarcoma	PET-CT/Bone scintigraphy/Thorax CT	11 / F
17	Hodgkin’s Lymphoma	PET-CT/ Thorax, abdominal and pelvic CT	11 / M
18	Ewing Sarcoma	PET-CT/Thorax CT	16 / M
19	Bone Lymphoma	Thorax, abdominal and pelvic CT	16 / M
20	PNET	Thorax, abdominal and pelvic CT	7 / F
21	Peritoneal Pseudomyxoma	Thorax, abdominal and pelvic CT	17 / M
22	Hodgkin’s Lymphoma	PET-CT/Thorax, abdominal and pelvic CT	17 / M
23	Alveolar Rhabdomyosarcoma	PET-CT/Cranial and thorax CT	17 / M
24	Wilms’ Tumor	Thorax, abdominal and pelvic CT	4 / M
25	Chondroblastoma	PET-CT/Bone scintigraphy	14 / M
26	Retinoblastoma	PET-CT	6 / F
27	Ganglioneuroblastoma	PET-CT/ Thorax CT	14 / F
28	Hodgkin’s Lymphoma	PET-CT	12 / M
29	Osteosarcoma	PET-CT/Bone scintigraphy/Thorax CT	18 / M
30	Osteosarcoma	PET-CT/Bone scintigraphy/Thorax CT	15 / M
31	Alveolar Rhabdomyosarcoma	PET-CT/Thorax CT	16 / F
32	Osteosarcoma	PET-CT/Bone scintigraphy/Thorax CT	6 / M

A total of 21 (61.8%) staging and 13 (38.2%) oncological follow-up procedures were performed (Table 3), of which 25 (73.5%) involved primary and/or secondary neoplasms, 13 (38%) presented local or locally advanced disease without evidence of distant metastases, 12 (35.2%) involved metastatic disease (including lymphoma), and nine (26.4%) involved follow-up patients without current disease according to radiological staging). Of the 16 patients with metastases at diagnosis, three had pulmonary metastases, seven had lymph node dissemination and three had bone metastases.

**Table 3:** Distribution according to demographics.

Parameters		Values
**Total cases**		32
**Total exams**		34
	Staging	21
	Follow up	13
**Age (years)**	Mean	13.3
	Median	14
**Gender**	Female	16
	Male	16

**Fig 1:**
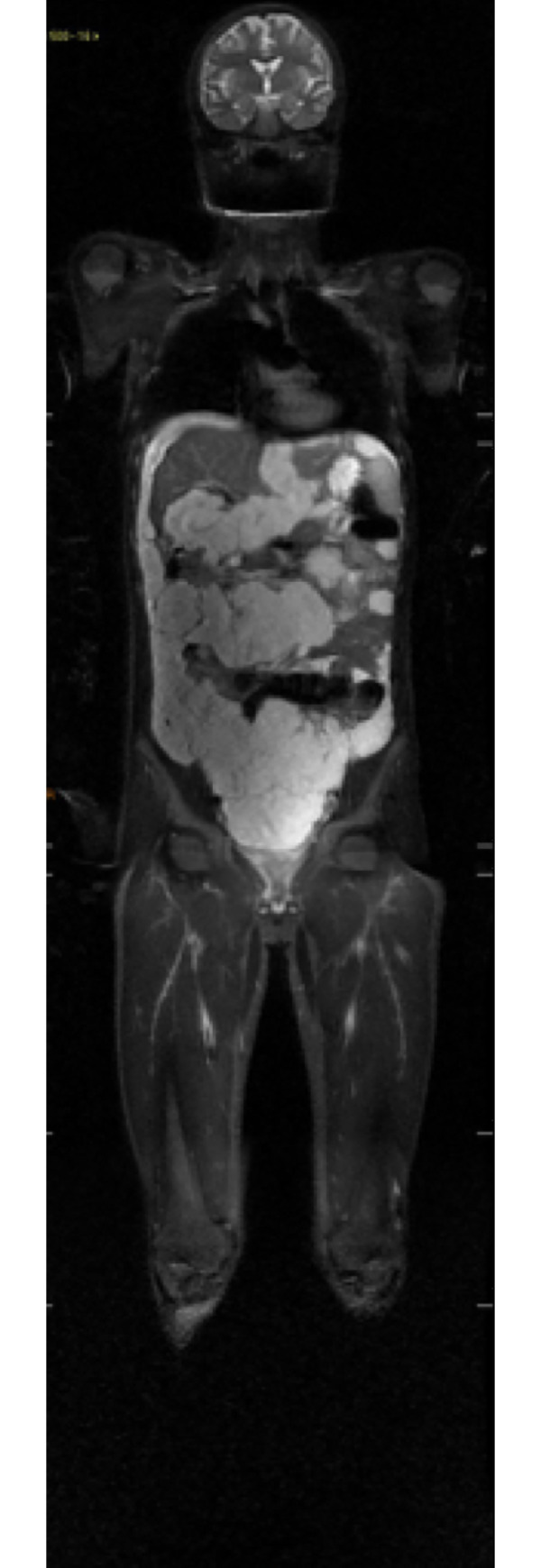
Coronal STIR sequence demonstrating multiple disseminated peritoneal lesions with high signal. No lesions were found at any other sites in this exam.

**Fig 2:**
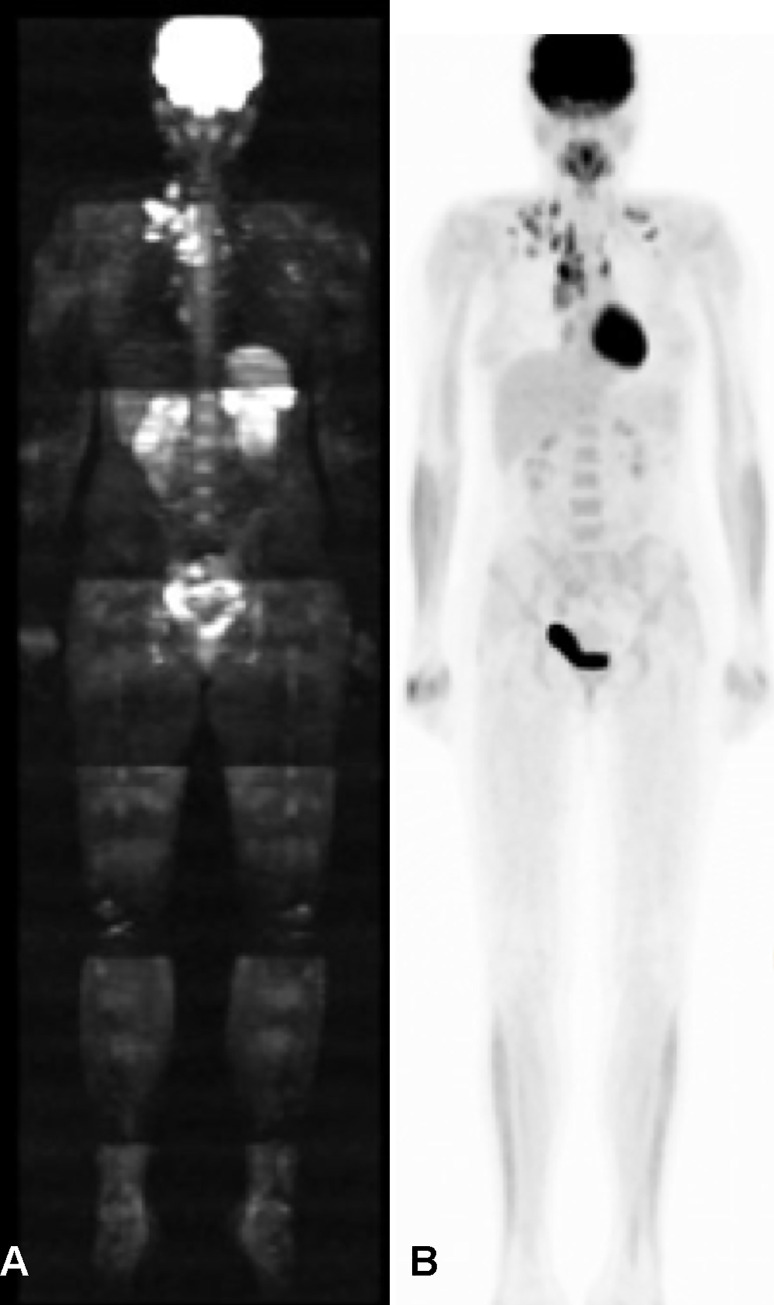
Female patient, fifteen-year-old, with Hodgkin's lymphoma, nodular sclerosis type. These images in the coronal plane, (A) diffusion and (B) PET-CT, show mediastinal and supraclavicular lymph node enlargement.

**Fig 3:**
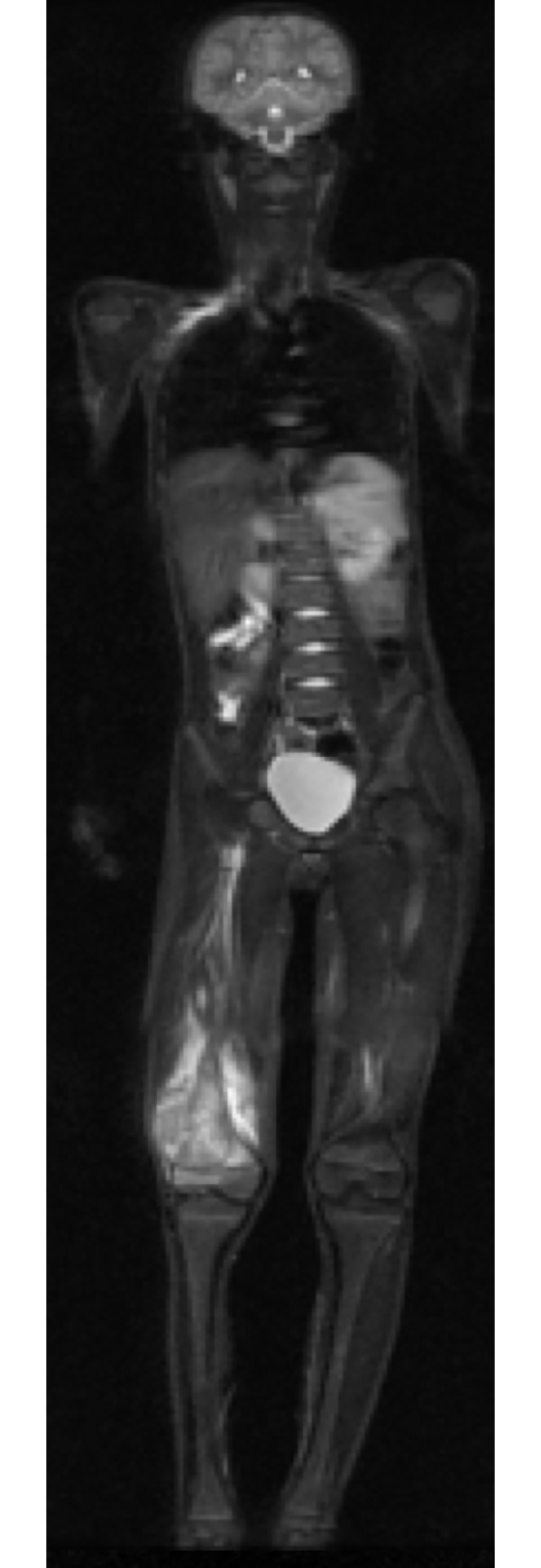
Male patient, thirteen-year-old, with osteoblastic osteosarcoma. (A) STIR image in the coronal plane showing large expansive formation in the right distal femur with infiltration of bone marrow and extension to adjacent soft tissues.

**Fig 4:**
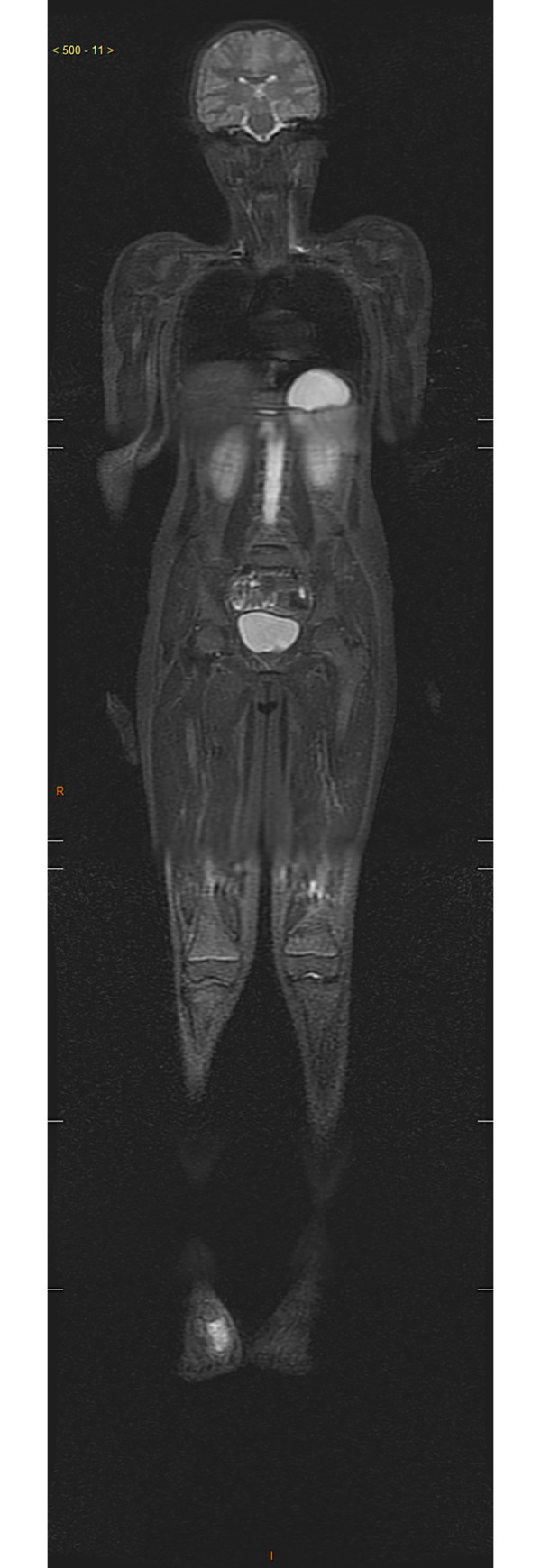
Female patient, eight-year-old, with chondrosarcoma. STIR image in the coronal plane showing the lesion in the right foot. There was no characterization of secondary lesions.

To further illustrate the aforementioned cases, we selected the following: an eighteen-year-old male patient with primary mucinous adenocarcinoma of the transverse colon and peritoneal pseudomyxoma (Fig 1), a fifteen-year-old female with Hodgkin's lymphoma, nodular sclerosis type (Fig 2), a thirteen-year-old male with osteoblastic osteosarcoma (Fig 3), an eight-year-old female with chondrosarcoma (Fig 4) and a sixteen-year-old male with Ewing's sarcoma with detectable lung metastasis in WBMRI. Further discussion about those cases is provided later.

The interobserver agreement for WBMRI was almost perfect for identifying the primary tumor (K = 0.947) and lung metastases (K = 1) and good for bone metastases (K = 0.727) and lymph node dissemination (K = 0.718).

Regarding the agreement between WBMRI after observer consensus and radiographic-clinical staging (Table 4), a perfect agreement was found in identifying the primary tumor, with WBMRI able to diagnose 100% of the cases (K = 1). There was also excellent agreement regarding bone metastases (K = 0.927). There was a good agreement for lymph node dissemination (K = 0.616) and moderate agreement for secondary pulmonary lesions (K = 0.477). Among the four patients whose lymphoma was being staged, there was excellent agreement (100%), with lymph node disease identified in two or more chains on the same side of the diaphragm (K = 1).

**Table 4:** Agreement between WBMRI and reference standard.

Variable	AGREEMENT
Primary tumor	Excellent K = 1
Bone metastasis	Excellent K = 0.927
Lymph node metastasis	Good K = 0.616
Pulmonary metastasis	Moderate K = 0.477

Regarding the primary tumor, we found a 100% sensitivity and specificity for WBMRI (PPV and NPV of 100%) in comparison to the reference standard. Regarding sites of secondary involvement, the sensitivity and specificity of WBMRI were the following: 100% and 90%, respectively, for the presence of bone metastases (PPV and NPV of 42% and 100%, respectively); 33% and 100% for the presence of pulmonary metastases (PPV and VPN 100% and 94%, respectively); and 85% and 100% for the presence of lymph node metastases (PPV and NPV of 100% and 96%, respectively).

When the presence or absence of primary or secondary neoplasia was investigated together, i.e., when patients with active neoplasm were separated from currently disease-free patients, we found a good correlation between WBMRI and the reference standard (k = 0.718), with WBMRI sensitivity and specificity of 100% and 62.5%, respectively (PPV and NPV of 89% and 100%, respectively, Table 5).

**Table 5:** WBMRI performance in relation to the reference standard.

Variable	Sensitivity	Specificity	PPV	NPV
Primary T	100	100	100	100
Bone metastasis	100	90	42	100
Lymph node metastasis	85	100	100	96
Pulmonary metastasis	33	100	100	94
Presence of neoplasia	100	62	89	100

As noted, we have obtained a sample with a variety of neoplasms, which demonstrated that WBMRI can detect different tumor types. There was a predominance of bone tumors, due to the fact that they are more common in adolescence, the age group in which we could perform the test without sedation. We do not that this high prevalence has interfered with the results.

Among the neoplasms, a case of peritoneal pseudomyxoma was observed in an eighteen-year-old male patient, with primary mucinous adenocarcinoma of the transverse colon, which is a rare clinical condition with an incidence of 1–2 cases per million and is characterized by the dissemination of mucinous implants on the peritoneal surface and by the progressive accumulation of gelatinous ascites (Fig 1). These findings contradict reports in the literature that it is more common in women in the fifth decade of life and is primarily located in the ovaries and appendix [14, 19–21].

We found an excellent interobserver agreement for WBMRI regarding the presence or absence of lesions at the different sites studied, which shows reproducibility of the results.

There was a perfect agreement between WBMRI and standard staging in identifying the primary tumor, with WBMRI able to diagnose 100% of the cases, which demonstrates that the method can diagnose different tumor types.

WBMRI could detect lymph node involvement in six of the seven cases in the sample, with no false positives (sensitivity 85% and specificity 100%). The patient who had a negative WBMRI result was diagnosed with a Wilms' tumor, including mesenteric and retroperitoneal lymph node compromise up to 14 mm. These dimensions, although above the cutoff (10 mm) commonly used to predict probable lymph node disease, are small and difficult to detect due to non-use of oral contrast, which would erase the fluid signal from intestinal loops, or even anti-peristaltic agents. Despite this, the results demonstrated good performance, thus agreeing with the findings of other authors [22, 23].

The sample included seven patients diagnosed with Hodgkin's lymphoma, four of whom were undergoing staging and had lymph node disease. Among these four patients, WBMRI identified lymph node disease in two or more chains on the same side of the diaphragm, a similar result to standard staging (Fig 2). The small sample limited precise evaluation of the exam’s role in this specific neoplasm, especially at other stages, but demonstrated excellent performance with the patients in this study (sensitivity and specificity 100%). This result agrees with the findings of Littooij et al., [23] who in 2014 published a prospective study of 36 children recently diagnosed with lymphoma, comparing WBMRI findings with PET-CT (gold standard) and finding sensitivity and specificity of 93% and 98%, respectively, for lymph node staging and 89% and 100%, respectively, for extranodal staging. They concluded that WBMRI is feasible and a good alternative for lymphoma staging. We also had a patient with primary bone lymphoma, whose disease was correctly detected by WBMRI.

Regarding bone lesions, the most frequent type observed, all 14 primary lesions were diagnosed precisely by WBMRI (Figs 3 and 4). There was 100% sensitivity in patients with bone metastases, although indeterminate lesions, whose standard staging suggested a benign nature, were detected in three patients. In one of these patients, who was diagnosed with Li-Fraumeni syndrome and had a history of multiple tumors (Hodgkin's lymphoma, a high-grade sarcoma on the left scapula, malignant fibrous histiocytoma, left adrenal carcinoma and three soft tissue sarcomas), bone lesions in the left clavicle and iliac lesions near the sacrum were indeterminate with WBMRI, although they demonstrated characteristics of low aggressiveness by standard staging and were considered probably benign. There was, however, no histological confirmation of the lesions. This patient underwent WBMRI a second time. Of the other two cases of false-positive WBMRI, one occurred while staging a retroperitoneal alveolar rhabdomyosarcoma, with indeterminate lesions in the proximal diaphysis of the right femur and the middle third of the right tibia. The other case occurred in retinoblastoma follow-up, with an altered distal metaphyseal signal in the right femur, which, as in the first case cited, presented an aspect of low aggressiveness and a lack of uptake during PET-CT. Both cases were being managed as benign lesions. Many studies in the literature agree with our results, showing extremely high sensitivity for WBMRI, many times higher than bone scintigraphy and similar to PET-CT [24–26]. In 2011, Wu et al., [27] published a meta-analysis involving 11 studies and 495 patients, finding excellent sensitivity and overall specificity WBMRI and concluded that it is certainly a tool with good accuracy, although the diffusion sequence seemed very sensitive but with low specificity.

**Fig 5:**
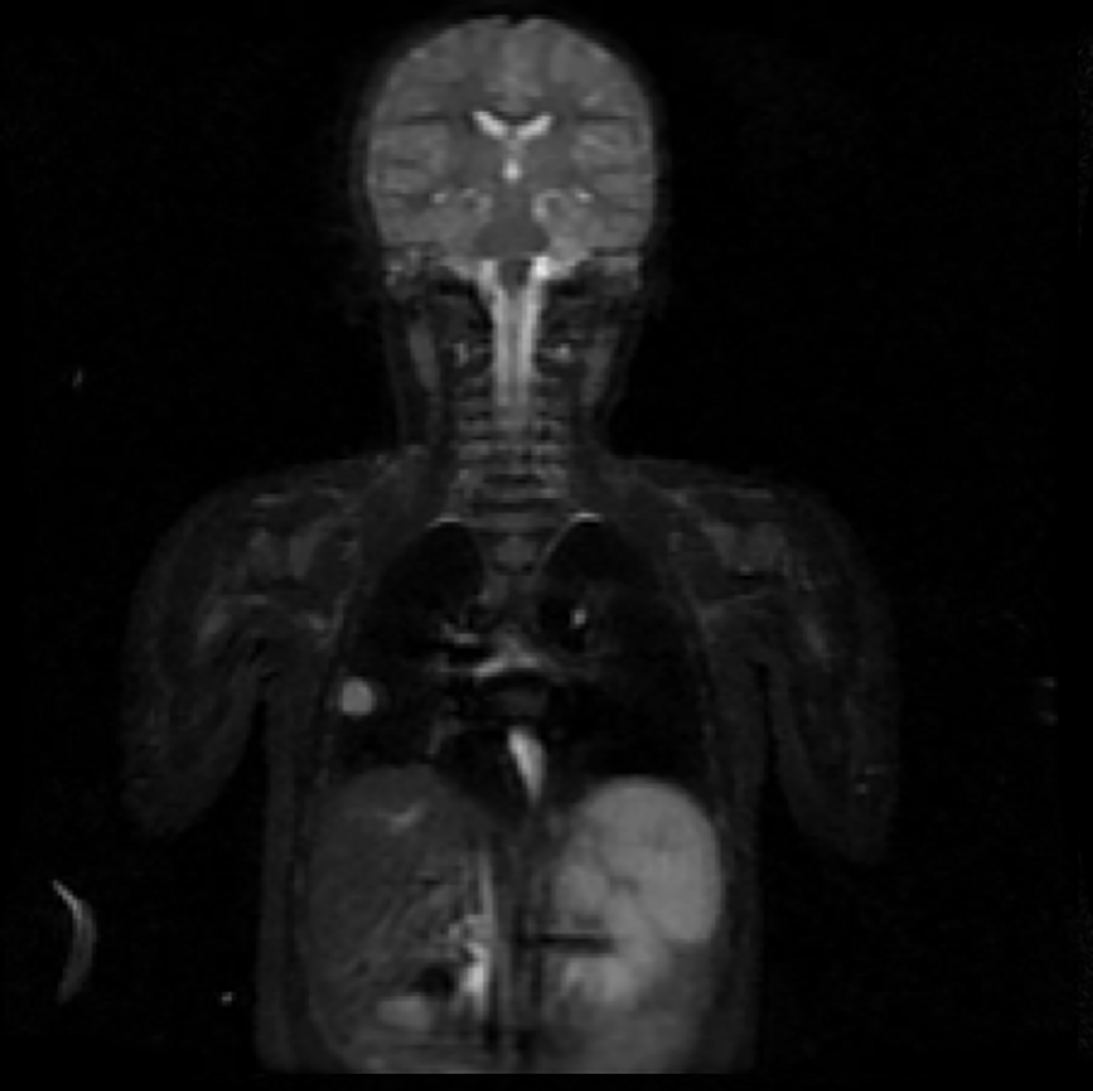
A well-characterized pulmonary nodule in a pediatric patient during follow up for Ewing's sarcoma.

We had three patients with secondary pulmonary lesions in the sample, and the presence of only one was detected by WBMRI (Fig 5). This low sensitivity differs from studies recently published in the literature [28–30] and could be explained by the small sample and by the size of the secondary pulmonary lesions, which were 12 mm or less in the two false-negative patients. Moreover, the tests were performed using the two-dimensional spin-echo sequence and not the volumetric three-dimensional gradient-echo sequence, which reduces spatial resolution and may impair the detection of subcentimetric pulmonary nodules.

When analyzing the presence or absence of primary or secondary neoplasia, i.e., separating patients with active neoplasms from currently disease-free patients, we found a good correlation between WBMRI and the reference standard (K = 0.718). In this circumstance, WBMRI was extremely sensitive (100%) and had acceptable specificity (62%). This feature denotes its great value as a screening test since patients with a negative result do not require further investigation with whole-body exams that use ionizing radiation.

Regarding the reference standard, we did not compare the WBMRI to any specific gold-standard imaging method, but instead, it was compared to the imaging assessment judged appropriated by the attendant physician. Also, as we aimed to compare the WBMRI with other ionizing radiation emitting imaging methods commonly offered, the researchers did not interfere in the decision-making process of the attendant physician in the sense of what tests were appropriated for each case.

The good interobserver agreement and consensus with traditional diagnostic methods, as well as the high sensitivity and acceptable specificity in a global investigation of neoplastic lesions, demonstrate that WBMRI can be an excellent qualitative method for patients without neoplastic disease. However, one exception to this would be subcentimetric pulmonary nodules, which, according to the results of this study, would be better assessed by low-dose chest CT, thus avoiding excessive exposure to ionizing radiation. For patients with positive WBMRI results, traditional radiological examinations are essential to precisely quantify and locate the lesions.

This study has certain limitations that should be considered. First, it had a small number of patients. Second, the exclusion of patients requiring sedation means that the sample did not reflect the actual incidence of major tumors in this pediatric population. Third, despite the excellent interobserver agreement, it was not possible to compare lesion-by-lesion detection in each patient, but only the presence or absence of a lesion in a certain organ or system, since due to the small sample we could not obtain symmetrical curves for the lesions to calculate the Kappa coefficient.

## Conclusions

Our results suggest that pediatric patients can be safely imaged with WBMRI, although not as the only tool but in association with low-dose chest CT (for subcentimeter pulmonary nodules). However, additional exams with ionizing radiation may be necessary for patients who tested positive to correctly quantify and locate the lesions.

## Supporting information

S1 Appendix (DOCX)

S1 File (ZIP)

## References

[1] Ahmed BA, Connolly BL, Shroff P, et al Cumulative effective doses from radiologic procedures for pediatric oncology patients. Pediatrics 2010; 126:e851–8. 10.1542/peds.2009-2675 20876178

[2] Chong AL, Grant RM, Ahmed BA, Thomas KE, Connolly BL, Greenberg M. Imaging in pediatric patients: time to think again about surveillance. Pediatr Blood Cancer 2010; 55:407–13. 10.1002/pbc.22575 20658609

[3] Granata C, Magnano G. Computerized tomography in pediatric oncology. Eur J Radiol 2013; 82:1098–107. 10.1016/j.ejrad.2011.12.008 22209525

[4] Kleinerman RA. Cancer risks following diagnostic and therapeutic radiation exposure in children. Pediatr Radiol 2006; 36 suppl 2:121–5. 16862418 10.1007/s00247-006-0191-5 PMC2663653

[5] Murano T, Tateishi U, Iinuma T, et al Evaluation of the risk of radiation exposure from new 18FDG PET/CT plans versus conventional X-ray plans in patients with pediatric cancers. Ann Nucl Med 2010; 24:261–7. 10.1007/s12149-010-0342-5 20237874

[6] Finelli DA. Diffusion-weighted imaging of acute vertebral compressions: specific diagnosis of benign versus malignant pathologic fractures. AJNR Am J Neuroradiol 2001; 22:241–2. 11156762 PMC7973958

[7] Herneth AM, Guccione S, Bednarski M. Apparent diffusion coefficient: a quantitative parameter for in vivo tumor characterization. Eur J Radiol 2003; 45:208–13. 10.1016/s0720-048x(02)00310-8 12595105

[8] Issa B. In vivo measurement of the apparent diffusion coefficient in normal and malignant prostatic tissues using echo-planar imaging. J Magn Reson Imaging 2002; 16:196–200. 10.1002/jmri.10139 12203768

[9] Low RN. Diffusion-weighted MR imaging for whole body metastatic disease and lymphadenopathy. Magn Reson Imaging Clin N Am 2009; 17:245–61. 10.1016/j.mric.2009.01.006 19406357

[10] Takeuchi M, Sasaki S, Ito M, et al Urinary bladder cancer: diffusion-weighted MR imaging—accuracy for diagnosing T stage and estimating histologic grade. Radiology 2009; 251:112–21. 10.1148/radiol.2511080873 19332849

[11] Tien RD, Felsberg GJ, Friedman H, Brown M, MacFall J. (3):671–7 MR imaging of high-grade cerebral gliomas: value of diffusion-weighted echoplanar pulse sequences. AJR Am J Roentgenol 1994; 162:671–7 10.2214/ajr.162.3.8109520 8109520

[12] Wang J, Takashima S, Takayama F, Kawakami S, Saito A, Matsushita T, Momose M, Ishiyama T. Head and neck lesions: characterization with diffusion-weighted echo-planar MR imaging. Radiology 2001; 220:621–30. 10.1148/radiol.2202010063 11526259

[13] Frat A, Ağildere M, Gençoğlu A, et al Value of whole-body turbo short tau inversion recovery magnetic resonance imaging with panoramic table for detecting bone metastases: comparison with 99MTc-methylene diphosphonate scintigraphy. J Comput Assist Tomogr 2006; 30:151–6. 10.1097/01.rct.0000189593.41198.20 16365592

[14] Namimoto T, Yamashita Y, Sumi S, Tang Y, Takahashi M. Focal liver masses: characterization with diffusion-weighted echo-planar MR imaging. Radiology 199; 204:739–44. 10.1148/radiology.204.3.9280252 9280252

[15] Siegel MJ, Acharyya S, Hoffer FA, et al Whole-body MR imaging for staging of malignant tumors in pediatric patients: results of the American College of Radiology Imaging Network 6660 Trial. Radiology 2013; 266:599–609. 10.1148/radiol.12112531 23264347 PMC3558875

[16] Gatidis S, Gückel B, la Fougère C, Schmitt J, Schäfer JF. [Simultaneous whole-body PET-MRI in pediatric oncology: More than just reducing radiation?]. Der Radiologe 2016; 56:62–30. 10.1007/s00117-016-0122-x 27306199

[17] Gatidis S, la Fougère C, Schaefer JF. Pediatric Oncologic Imaging: A Key Application of Combined PET/MRI. RoFo 2016; 188:359–64. 10.1055/s-0041-109513 27002497

[18] Pugmire BS, Guimaraes AR, Lim R, et al Simultaneous whole body (18) F-fluorodeoxyglucose positron emission tomography magnetic resonance imaging for evaluation of pediatric cancer: Preliminary experience and comparison with (18)F-fluorodeoxyglucose positron emission tomography computed tomograp. World J Radiol 2016; 8:322–30. 10.4329/wjr.v8.i3.322 27028112 PMC4807342

[19] Chauhan A, Patodi N, Ahmed M. A rare cause of ascites: pseudomyxoma peritonei and a review of the literature. Clin Cases Rep 2015; 3:156–9. 10.1002/ccr3.188 PMC4377246 25838904

[20] Guo A, Li Y, Wei L. Pseudomyxoma peritonei of 92 chinese patients: Clinical characteristics, pathological classification and prognostic factors. Word J Gastroenterol 2012; 18:3081–8. 10.3748/wjg.v18.i24.3081 PMC3386321 22791943

[21] Mavrodin CJ, Pariza G, Iordache P, Sajin M. Pseudomyxoma peritonei, a rare entity difficult to diagnose and treat–case report. Chirurgia 2014; 109:846–9. 25560512

[22] Brennan DD., Gleeson T, Coate E, Cronin C, Carney D, Eustace SJ. A Comparison of Whole-Body MRI and CT for the Staging of Lymphoma. Am J Roentgenol 2005; 185:711–6. 16120924 10.2214/ajr.185.3.01850711

[23] Littooij AS, Kwee TC, Barber I, et al Whole-body MRI for initial staging of paediatric lymphoma: Prospective comparison to an FDG-PET/CT-based reference standard. Eur Radiol 2014; 24:1153–65. 10.1007/s00330-014-3114-0 24563179

[24] Cascini G, Falcone C, Greco C, Bertucci B, Cipullo S, Tamburrini O. Whole-body magnetic resonance imaging for detecting bone metastases: comparison with bone scintigraphy. Radiol Med 2008; 113:1157–70. 10.1007/s11547-008-0341-y 18958408

[25] Ghanem N, Uhl M, Brink I, et al Diagnostic value of MRI in comparison to scintigraphy, PET, MS-CT and PET/CT for the detection of metastases of bone. Eur J Radiol 2005; 55:41–55. 10.1016/j.ejrad.2005.01.016 15950100

[26] Schmidt GP, Schoenberg SO, Schmid R, et al Screening for bone metastases: whole-body MRI using a 32-channel system versus dual-modality PET-CT. Eur Radiol 2007; 17:939–49. 10.1007/s00330-006-0361-8 16951929

[27] Wu L-M, Gu H-Y, Zheng J, et al Diagnostic value of whole-body magnetic resonance imaging for bone metastases: a systematic review and meta-analysis. J Magn Reson Imaging 2011; 34:128–35. 10.1002/jmri.22608 21618333

[28] Hochhegger B, Marchiori E, Sedlaczek O, et al MRI in lung cancer: a pictorial essay. Br J Radiol 2011; 84:661–8. 10.1259/bjr/24661484 21697415 PMC3473490

[29] Kurihara Y, Matsuoka S, Yamashiro T, et al MRI of pulmonary nodules. Am J Roentgenol 2014; 202:W210–6. 24555616 10.2214/AJR.13.11618

[30] Wang YX, Lo GG, Yuan J, Larson PE, Zhang X. Magnetic resonance imaging for lung cancer screen. J Thorac Dis 2014; 6:1340–8. 10.3978/j.issn.2072-1439.2014.08.43 25276380 PMC4178109

