# Supplementary material for: Evaluation of whole-body MRI with diffusion-weighted sequences in the staging of pediatric cancer patients
Source: PLoS One. 2020 Aug 27;15(8):e0238166. doi: 10.1371/journal.pone.0238166 (PMC7451574; doi:10.1371/journal.pone.0238166)
Supplement: S1 Appendix — (DOCX) [file pone.0238166.s001.docx]

**S1 Appendix**

Detail on imaging methods protocol:

*Whole-body MRI protocol*

Performed with a 1.5 Tesla MR imager (Signa Excite HD; GE Healthcare, Milwaukee, USA) with a body coil and maximal gradient power of 33 mT/m and a pulse rate of 160 mT/m/s.

| **Parameter** | **T1 Spin-echo axial** | **STIR coronal** | **DW Echo-planar imaging axial** |
| --- | --- | --- | --- |
| TR | 370 ms | 5420 ms | 1800 ms |
| TE | 15 ms | 66 ms | 93,8 ms |
| Matrix | 512 x 512 | 512 x 512 | 160 x 192 |
| FOV | 340 mm | 515 mm | 360 mm |
| NEX | 1 |  | 16 |
| Slice thickness/Gap | 5 mm/1 mm | 7 mm/0 | 5 mm/0 |
| B-value |  |  | 0 – 600 s/mm² |

The acquired images were transferred to a workstation (Advantage Windows version 4.2_07; GE Healthcare, Milwaukee, USA) and the DW acquisition was post-processed with commercial software (Functool; GE Healthcare, Milwaukee, EUA).

- CT scan protocol:

Performed in a 16-channel scanner (Philips Brilliance Big Bore).

| **Slice thickness** | 3 mm |
| --- | --- |
| **Gap** | 1,5 mm |
| **KVp** | 80 |
| **mAs** | Dynamic modulating system calculated (Dose right Down) |
| **Contrast dose** | 1-2 mL per kilogram |

*PET-scan protocol*

Performed in a 16-channel scanner (GE Discovery 600) after the administration of ^18^F-FDG (0,12 mCi per kilogram) following a minimal interval of 60-120 minutes after dose administration.

CT slices were acquired with a helicoidal low-dosage technique (Slice thickness = 2,5 mm; Gap = 0), without administration of intravenous contrast interesting all body segments (head and neck, thorax, abdomen and pelvis, lower limbs).

The standard uptake values (SUV) were calculated for clinically relevant areas and imaging analysis was performed in a dedicated workstation (Philips – EBW using an imaging fusion software).
