# Supplementary material for: Evaluation of whole-body MRI with diffusion-weighted sequences in the staging of pediatric cancer patients
Source: PLoS One. 2020 Aug 27;15(8):e0238166. doi: 10.1371/journal.pone.0238166 (PMC7451574; doi:10.1371/journal.pone.0238166)
Supplement: S1 File — (ZIP) [file pone.0238166.s002.zip › DADOS_ADRENAL_REVER.pdf]

```
FREQUENCIES VARIABLES=nódulo_adrenal_rmci_1 nódulo_adrenal_rmci_2 nódulo_a
drenal_estad_padrão nódulo_adrenal_conseno_rmci nódulo_adr
   enal_estad_clínico_radiológico
  /ORDER=ANALYSIS.
```

Frequencies

Notes

	Output Created	15-Nov-2016 20h3min44s
	Comments	
Input	Data	C:\Users\Fábio\Desktop\ALEX_SPSS\PLANILHA.sav
	Active Dataset	DataSet1
	Filter	<none>
	Weight	<none>
	Split File	<none>
	N of Rows in Working Data File	34
Missing Value Handling	Definition of Missing	User-defined missing values are treated as missing.
	Cases Used	Statistics are based on all cases with valid data.
	Syntax	FREQUENCIES VARIABLES=nódulo_adrenal_rmci_1 nódulo_adrenal_rmci_2 nódulo_adrenal_estad_padrão nódulo_adrenal_conseno_rmci nódulo_adrenal_estad_clínico_radiológico /ORDER=ANALYSIS.
Resources	Processor Time	0:00:00.016
	Elapsed Time	0:00:00.016

```
[DataSet1] C:\Users\Fábio\Desktop\ALEX_SPSS\PLANILHA.sav
```

Statistics

		nódulo_adrenal_rmci_1	nódulo_adrenal_rmci_2	nódulo_adrenal_estad_padrão	nódulo_adrenal_conseno_rmci	nódulo_adrenal_estad_clínico_radiológico
N	Valid	34	34	34	34	34
	Missing	0	0	0	0	0

Frequency Table

nódulo_adrenal_rmci_1

		Frequency	Percent	Valid Percent	Cumulative Percent
Valid	AUSENTE	33	97,1	97,1	97,1
	PRESENTE	1	2,9	2,9	100,0
	Total	34	100,0	100,0	

nódulo_adrenal_rmci_2

		Frequency	Percent	Valid Percent	Cumulative Percent
Valid	AUSENTE	34	100,0	100,0	100,0

nódulo_adrenal_estad_padrão

		Frequency	Percent	Valid Percent	Cumulative Percent
Valid	AUSENTE	34	100,0	100,0	100,0

nódulo_adrenal_conseno_rmci

		Frequency	Percent	Valid Percent	Cumulative Percent
Valid	AUSENTE	33	97,1	97,1	97,1
	PRESENTE	1	2,9	2,9	100,0
	Total	34	100,0	100,0	

nódulo_adrenal_estad_clínico_radiológico

		Frequency	Percent	Valid Percent	Cumulative Percent
Valid	AUSENTE	34	100,0	100,0	100,0

```
CROSSTABS
  /TABLES=nódulo_adrenal_conseno_rmci BY nódulo_adrenal_estad_clínico_radi
ológico
  /FORMAT=AVALUE TABLES
  /STATISTICS=KAPPA
  /CELLS=COUNT TOTAL
  /COUNT ROUND CELL.
```

Crosstabs

Notes

	Output Created	15-Nov-2016 20h4min14s
	Comments	
Input	Data	C:\Users\Fábio\Desktop\ALEX_ SPSS\PLANILHA.sav
	Active Dataset	DataSet1
	Filter	<none>
	Weight	<none>
	Split File	<none>
	N of Rows in Working Data File	34
Missing Value Handling	Definition of Missing	User-defined missing values are treated as missing.
	Cases Used	Statistics for each table are based on all the cases with valid data in the specified range(s) for all variables in each table.

Notes

	Syntax	CROSSTABS<br>/TABLES=nódulo_adrenal_conseno_rmci BY nódulo_adrenal_estad_clínico_radiológico<br>/FORMAT=AVALUE TABLES<br>/STATISTICS=KAPPA<br>/CELLS=COUNT TOTAL<br>/COUNT ROUND CELL.
Resources	Processor Time	0:00:00.015
	Elapsed Time	0:00:00.003
	Dimensions Requested	2
	Cells Available	174762

```
[DataSet1] C:\Users\Fábio\Desktop\ALEX_SPSS\PLANILHA.sav
```

Warnings

No measures of association are computed for the crosstabulation of nódulo_adrenal_conseno_rmci * nódulo_adrenal_estad_clínico_radiológico. At least one variable in each 2-way table upon which measures of association are computed is a constant.

Case Processing Summary

	Cases					
	Valid		Missing		Total	
	N	Percent	N	Percent	N	Percent
nódulo_adrenal_conseno_rmci * nódulo_adrenal_estad_clínico_radiológico	34	100,0%	0	,0%	34	100,0%

nódulo_adrenal_conseno_rmci * nódulo_adrenal_estad_clínico_radiológico Crosstabulation

			nódulo_adrenal_estad_clínico_radiológico	
			AUSENTE	Total
nódulo_adrenal_conseno_rmci	AUSENTE	Count	33	33
		% of Total	97,1%	97,1%
	PRESENTE	Count	1	1
		% of Total	2,9%	2,9%
	Total	Count	34	34
		% of Total	100,0%	100,0%

Symmetric Measures

		Value
Measure of Agreement	Kappa	.[a]
	N of Valid Cases	34

a. No statistics are computed because nódulo_adrenal_estad_clínico_radiológico is a constant.
