# Supplementary material for: Evaluation of whole-body MRI with diffusion-weighted sequences in the staging of pediatric cancer patients
Source: PLoS One. 2020 Aug 27;15(8):e0238166. doi: 10.1371/journal.pone.0238166 (PMC7451574; doi:10.1371/journal.pone.0238166)
Supplement: S1 File — (ZIP) [file pone.0238166.s002.zip › DADOS_COMPROMETIMENTO_LINFONODAL.pdf]

```
FREQUENCIES VARIABLES=comprometimento_linfonodal_primário_RMCI_1 compromet
imento_linfonodal_primário_RMC1_2 comprometimento_linfonod
   al_primário_estad_padrão comprometimento_linfonodal_primário_estad_clín
ico_radiológico
comprometimento_linfonodal_primário_consenso_rmci
  /ORDER=ANALYSIS.
```

Frequencies

Notes

	Output Created	15-Nov-2016 19h55min46s
	Comments	
Input	Data	C:\Users\Fábio\Desktop\ALEX_SPSS\PLANILHA.sav
	Active Dataset	DataSet1
	Filter	<none>
	Weight	<none>
	Split File	<none>
	N of Rows in Working Data File	34
Missing Value Handling	Definition of Missing	User-defined missing values are treated as missing.
	Cases Used	Statistics are based on all cases with valid data.
	Syntax	FREQUENCIES VARIABLES=comprometimento_linfonodal_primário_RMCI_1 comprometimento_linfonodal_primário_RMC1_2 comprometimento_linfonodal_primário_estad_padrão comprometimento_linfonodal_primário_estad_clínico_radiológico comprometimento_linfonodal_primário_consenso_rmci /ORDER=ANALYSIS.
Resources	Processor Time	0:00:00.000
	Elapsed Time	0:00:00.000

```
[DataSet1] C:\Users\Fábio\Desktop\ALEX_SPSS\PLANILHA.sav
```

Statistics

		comprometimento_linfonodal_primário_RMCI_1	comprometimento_linfonodal_primário_RMC1_2	comprometimento_linfonodal_primário_estad_padrão	comprometimento_linfonodal_primário_estad_clínico_radiológico	comprometimento_linfonodal_primário_consenso_rmci
N	Valid	34	34	34	34	34
	Missing	0	0	0	0	0

Frequency Table

comprometimento_linfonodal_primário_RMCI_1

		Frequency	Percent	Valid Percent	Cumulative Percent
Valid	AUSENTE	3	8,8	8,8	8,8
	2,00	4	11,8	11,8	20,6
	100,00	27	79,4	79,4	100,0
	Total	34	100,0	100,0	

comprometimento_linfonodal_primário_RMC1_2

		Frequency	Percent	Valid Percent	Cumulative Percent
Valid	AUSENTE	3	8,8	8,8	8,8
	2,00	4	11,8	11,8	20,6
	100,00	27	79,4	79,4	100,0
	Total	34	100,0	100,0	

comprometimento_linfonodal_primário_estad_padrão

		Frequency	Percent	Valid Percent	Cumulative Percent
Valid	AUSENTE	3	8,8	8,8	8,8
	2,00	4	11,8	11,8	20,6
	100,00	27	79,4	79,4	100,0
	Total	34	100,0	100,0	

comprometimento_linfonodal_primário_estad_clínico_radiológico

		Frequency	Percent	Valid Percent	Cumulative Percent
Valid	AUSENTE	3	8,8	8,8	8,8
	2,00	4	11,8	11,8	20,6
	100,00	27	79,4	79,4	100,0
	Total	34	100,0	100,0	

comprometimento_linfonodal_primário_consenso_rmci

		Frequency	Percent	Valid Percent	Cumulative Percent
Valid	AUSENTE	3	8,8	8,8	8,8
	2,00	4	11,8	11,8	20,6
	100,00	27	79,4	79,4	100,0
	Total	34	100,0	100,0	

```
CROSSTABS
  /TABLES=comprometimento_linfonodal_primário_consenso_rmci BY comprometim
ento_linfonodal_primário_estad_clínico_radiológico
  /FORMAT=AVALUE TABLES
  /STATISTICS=KAPPA
  /CELLS=COUNT
  /COUNT ROUND CELL.
```

Crosstabs

Notes

	Output Created	15-Nov-2016 19h56min25s
	Comments	
Input	Data	C:\Users\Fábio\Desktop\ALEX_ SPSS\PLANILHA.sav
	Active Dataset	DataSet1
	Filter	<none>
	Weight	<none>
	Split File	<none>
	N of Rows in Working Data File	34
Missing Value Handling	Definition of Missing	User-defined missing values are treated as missing.
	Cases Used	Statistics for each table are based on all the cases with valid data in the specified range(s) for all variables in each table.
	Syntax	CROSSTABS /TABLES=comprometimento_ linfonodal_primário_consenso_ rmci BY comprometimento_linfonodal_ primário_estad_clínico_radiológico /FORMAT=AVALUE TABLES /STATISTICS=KAPPA /CELLS=COUNT /COUNT ROUND CELL.
Resources	Processor Time	0:00:00.016
	Elapsed Time	0:00:00.015
	Dimensions Requested	2
	Cells Available	174762

```
[DataSet1] C:\Users\Fábio\Desktop\ALEX_SPSS\PLANILHA.sav
```

Case Processing Summary

	Cases					
	Valid		Missing		Total	
	N	Percent	N	Percent	N	Percent
comprometimento_ linfonodal_primário_ consenso_rmci * comprometimento_ linfonodal_primário_ estad_clínico_radiológico	34	100,0%	0	,0%	34	100,0%

comprometimento_linfonodal_primário_consenso_rmci * comprometimento_linfonodal_primário_estad_clínico_radiológico Crosstabulation

Count

		comprometimento_linfonodal_primário_estad_ clínico_radiológico			
		AUSENTE	2,00	100,00	Total
comprometimento_ linfonodal_primário_ consenso_rmci	AUSENTE	3	0	0	3
	2,00	0	4	0	4

comprometimento_linfonodal_primário_consenso_rmci * comprometimento_linfonodal_primário_estad_clínico_radiológico Crosstabulation

Count

		comprometimento_linfonodal_primário_estad_clínico_radiológico			
		AUSENTE	2,00	100,00	Total
comprometimento_linfonodal_primário_consenso_rmci	100,00	0	0	27	27
	Total	3	4	27	34

Symmetric Measures

		Value	Asymp. Std. Error[a]	Approx. T[b]	Approx. Sig.
Measure of Agreement	Kappa	1,000	,000	7,585	,000
	N of Valid Cases	34			

a. Not assuming the null hypothesis.

b. Using the asymptotic standard error assuming the null hypothesis.

```
CROSSTABS
  /TABLES=comprometimento_linfonodal_primário_consenso_rmci BY comprometim
ento_linfonodal_primário_estad_clínico_radiológico
  /FORMAT=AVALUE TABLES
  /STATISTICS=KAPPA
  /CELLS=COUNT TOTAL
  /COUNT ROUND CELL.
```

Crosstabs

Notes

	Output Created	15-Nov-2016 19h56min56s
	Comments	
Input	Data	C:\Users\Fábio\Desktop\ALEX_SPSS\PLANILHA.sav
	Active Dataset	DataSet1
	Filter	<none>
	Weight	<none>
	Split File	<none>
	N of Rows in Working Data File	34
Missing Value Handling	Definition of Missing	User-defined missing values are treated as missing.
	Cases Used	Statistics for each table are based on all the cases with valid data in the specified range(s) for all variables in each table.

Notes

	Syntax	CROSSTABS<br>/TABLES=comprometimento_linfonodal_primário_consenso_rmci BY comprometimento_linfonodal_primário_estad_clínico_radiológico<br>/FORMAT=AVALUE TABLES<br>/STATISTICS=KAPPA<br>/CELLS=COUNT TOTAL<br>/COUNT ROUND CELL.
Resources	Processor Time	0:00:00.000
	Elapsed Time	0:00:00.000
	Dimensions Requested	2
	Cells Available	174762

```
[DataSet1] C:\Users\Fábio\Desktop\ALEX_SPSS\PLANILHA.sav
```

Case Processing Summary

	Cases					
	Valid		Missing		Total	
	N	Percent	N	Percent	N	Percent
comprometimento_linfonodal_primário_consenso_rmci * comprometimento_linfonodal_primário_estad_clínico_radiológico	34	100,0%	0	,0%	34	100,0%

comprometimento_linfonodal_primário_consenso_rmci * comprometimento_linfonodal_primário_estad_clínico_radiológico Crosstabulation

			comprometimento_linfonodal_primário_estad_clínico_radiológico	
			AUSENTE	2,00
comprometimento_linfonodal_primário_consenso_rmci	AUSENTE	Count	3	0
		% of Total	8,8%	,0%
	2,00	Count	0	4
		% of Total	,0%	11,8%
	100,00	Count	0	0
		% of Total	,0%	,0%
Total		Count	3	4
		% of Total	8,8%	11,8%

comprometimento_linfonodal_primário_consenso_rmci * comprometimento_linfonodal_primário_estad_clínico_radiológico Crosstabulation

			comprometimento_linfonodal_primário_estad_clínico_radiológico	
			100,00	Total
comprometimento_linfonodal_primário_consenso_rmci	AUSENTE	Count	0	3
		% of Total	,0%	8,8%
	2,00	Count	0	4
		% of Total	,0%	11,8%
	100,00	Count	27	27
		% of Total	79,4%	79,4%
	Total	Count	27	34
		% of Total	79,4%	100,0%

Symmetric Measures

		Value	Asymp. Std. Error[a]	Approx. T[b]	Approx. Sig.
Measure of Agreement	Kappa	1,000	,000	7,585	,000
	N of Valid Cases	34			

a. Not assuming the null hypothesis.

b. Using the asymptotic standard error assuming the null hypothesis.
