# Supplementary material for: Evaluation of whole-body MRI with diffusion-weighted sequences in the staging of pediatric cancer patients
Source: PLoS One. 2020 Aug 27;15(8):e0238166. doi: 10.1371/journal.pone.0238166 (PMC7451574; doi:10.1371/journal.pone.0238166)
Supplement: S1 File — (ZIP) [file pone.0238166.s002.zip › DADOS_DEMOGR╡FICOS.pdf]

```
SAVE OUTFILE='C:\Users\Fábio\Desktop\ALEX_SPSS\PLANILHA.sav'
 /COMPRESSED.
FREQUENCIES VARIABLES=QUALIFICAÇÃO diagnóstico sexo
  /ORDER=ANALYSIS.
```

Frequencies

Notes

	Output Created	15-Nov-2016 16h10min24s
	Comments	
Input	Data	C:\Users\Fábio\Desktop\ALEX_SPSS\PLANILHA.sav
	Active Dataset	DataSet1
	Filter	<none>
	Weight	<none>
	Split File	<none>
	N of Rows in Working Data File	34
Missing Value Handling	Definition of Missing	User-defined missing values are treated as missing.
	Cases Used	Statistics are based on all cases with valid data.
	Syntax	FREQUENCIES VARIABLES=QUALIFICAÇÃO diagnóstico sexo /ORDER=ANALYSIS.
Resources	Processor Time	0:00:00.016
	Elapsed Time	0:00:00.006

```
[DataSet1] C:\Users\Fábio\Desktop\ALEX_SPSS\PLANILHA.sav
```

Statistics

		QUALIFICAÇÃO	diagnóstico	sexo
N	Valid	34	34	34
	Missing	0	0	0

Frequency Table

QUALIFICAÇÃO

		Frequency	Percent	Valid Percent	Cumulative Percent
Valid	ESTADIAMENTO	21	61,8	61,8	61,8
	SEGUIMENTO	13	38,2	38,2	100,0
	Total	34	100,0	100,0	

diagnóstico

		Frequency	Percent	Valid Percent	Cumulative Percent
Valid	rabdomiossarcoma embrionário variante esclerosante	3	8,8	8,8	8,8
	osteossarcoma	12	35,3	35,3	44,1
	TCG de Seio endodérmico sacrococcígeo	1	2,9	2,9	47,1
	linfoma Hodgkin	6	17,6	17,6	64,7
	Lifraumeni, com múltiplos tumores primários	2	5,9	5,9	70,6
	sarcoma de Ewing	1	2,9	2,9	73,5
	linfoma linfoblástico ósseo	1	2,9	2,9	76,5
	pnet	1	2,9	2,9	79,4
	pseudomixoma peritoneal	1	2,9	2,9	82,4
	tumor de Wilms	1	2,9	2,9	85,3
	condroblastoma	1	2,9	2,9	88,2
	retinoblastoma	1	2,9	2,9	91,2
	ganglioneuroblastoma:	1	2,9	2,9	94,1
	neoplasia ovariana	2	5,9	5,9	100,0
	Total	34	100,0	100,0	

sexo

		Frequency	Percent	Valid Percent	Cumulative Percent
Valid	PRESENTE	17	50,0	50,0	50,0
	2,00	17	50,0	50,0	100,0
	Total	34	100,0	100,0	

```
EXAMINE VARIABLES=idade
  /PLOT BOXPLOT STEMLEAF NPPLOT
  /COMPARE GROUP
  /STATISTICS DESCRIPTIVES
  /CINTERVAL 95
  /MISSING LISTWISE
  /NOTOTAL.
```

Explore

Notes

Output Created	15-Nov-2016 16h11min21s
Comments	

Notes

Input	Data	C:\Users\Fábio\Desktop\ALEX_SPSS\PLANILHA.sav
	Active Dataset	DataSet1
	Filter	<none>
	Weight	<none>
	Split File	<none>
	N of Rows in Working Data File	34
Missing Value Handling	Definition of Missing	User-defined missing values for dependent variables are treated as missing.
	Cases Used	Statistics are based on cases with no missing values for any dependent variable or factor used.
	Syntax	EXAMINE VARIABLES=idade /PLOT BOXPLOT STEMLEAF NPPLOT /COMPARE GROUP /STATISTICS DESCRIPTIVES /CINTERVAL 95 /MISSING LISTWISE /NOTOTAL.
Resources	Processor Time	0:00:01.110
	Elapsed Time	0:00:01.150

```
[DataSet1] C:\Users\Fábio\Desktop\ALEX_SPSS\PLANILHA.sav
```

Case Processing Summary

	Cases					
	Valid		Missing		Total	
	N	Percent	N	Percent	N	Percent
idade	34	100,0%	0	,0%	34	100,0%

Descriptives

			Statistic	Std. Error
idade		Mean	13,03	,786
	95% Confidence Interval for Mean	Lower Bound	11,43	
		Upper Bound	14,63	
		5% Trimmed Mean	13,29	
		Median	14,00	
		Variance	20,999	
		Std. Deviation	4,582	
		Minimum	3	
		Maximum	18	
		Range	15	
		Interquartile Range	7	
		Skewness	-,721	,403
		Kurtosis	-,615	,788

Tests of Normality

	Kolmogorov-Smirnov[a]			Shapiro-Wilk		
	Statistic	df	Sig.	Statistic	df	Sig.
idade	,183	34	,006	,887	34	,002

a. Lilliefors Significance Correction

idade

```
idade Stem-and-Leaf Plot

 Frequency    Stem &  Leaf

     1,00        0 .  3
     2,00        0 .  45
     3,00        0 .  667
      ,00        0 .
     7,00        1 .  0001111
     3,00        1 .  233
     3,00        1 .  445
    10,00        1 .  6667777777
     5,00        1 .  88888

 Stem width:        10
 Each leaf:       1 case(s)
```

Normal Q-Q Plot of idade

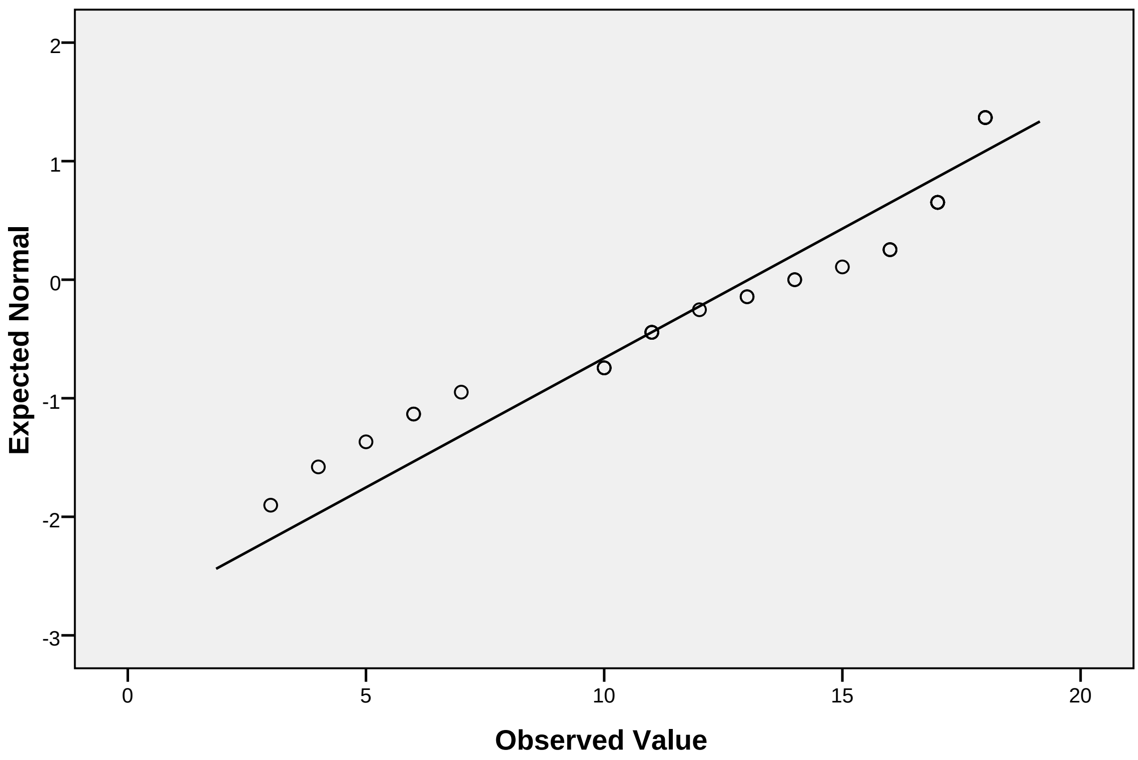

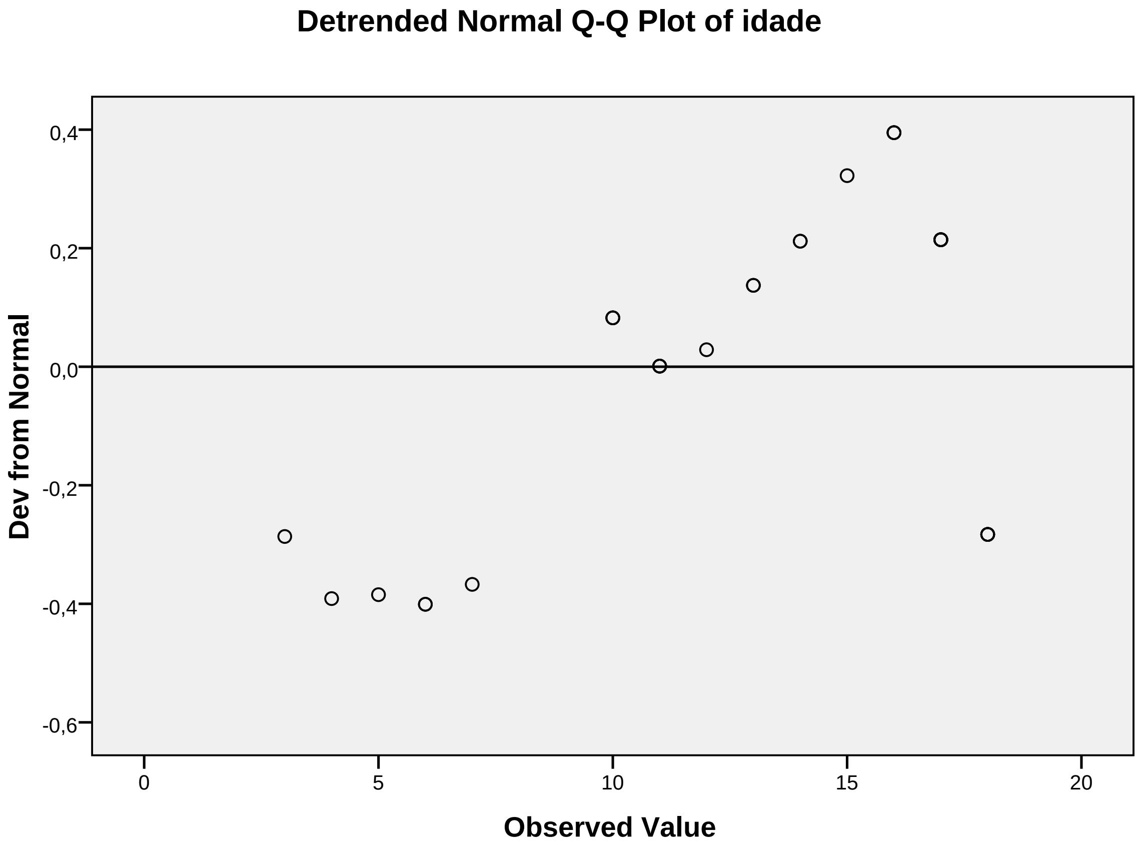
Detrended Normal Q-Q Plot of idade
Dev from Normal
0,4
0,2
0,0
-0,2
-0,4
-0,6
0
5
10
15
20
Observed Value

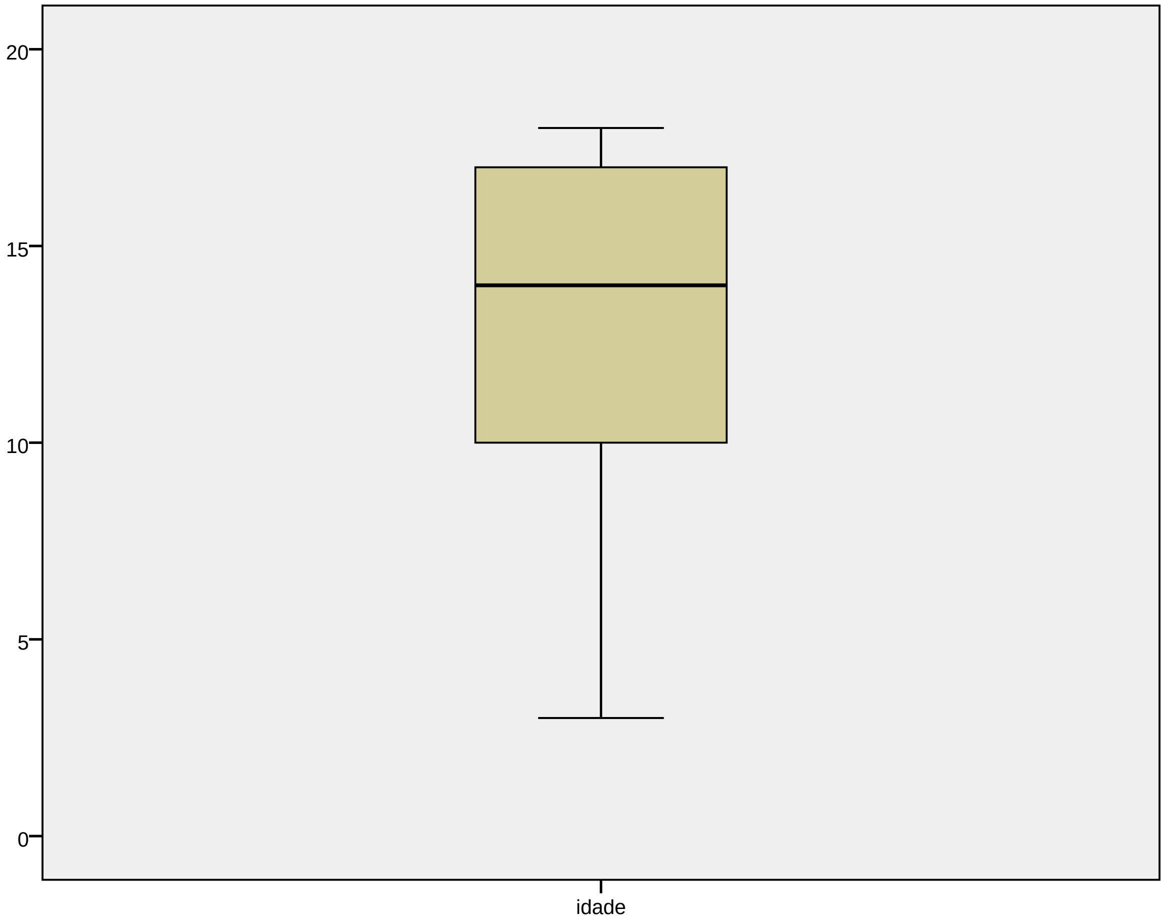
20
15
10
5
0
idade
