# Supplementary material for: Evaluation of whole-body MRI with diffusion-weighted sequences in the staging of pediatric cancer patients
Source: PLoS One. 2020 Aug 27;15(8):e0238166. doi: 10.1371/journal.pone.0238166 (PMC7451574; doi:10.1371/journal.pone.0238166)
Supplement: S1 File — (ZIP) [file pone.0238166.s002.zip › DADOS_DERRAME_PLEURAL.pdf]

```
FREQUENCIES VARIABLES=derrame_pleural_RMCI_1 derrame_pleural_RMCI_2 derram
e_pleural_estad_padrão derrame_pleural_consenso_RMCI derra
   me_pleural_estad_clínico_radiológico
  /ORDER=ANALYSIS.
```

Frequencies

Notes

	Output Created	15-Nov-2016 19h54min3s
	Comments	
Input	Data	C:\Users\Fábio\Desktop\ALEX_SPSS\PLANILHA.sav
	Active Dataset	DataSet1
	Filter	<none>
	Weight	<none>
	Split File	<none>
	N of Rows in Working Data File	34
Missing Value Handling	Definition of Missing	User-defined missing values are treated as missing.
	Cases Used	Statistics are based on all cases with valid data.
	Syntax	FREQUENCIES VARIABLES=derrame_pleural_RMCI_1 derrame_pleural_RMCI_2 derrame_pleural_estad_padrão derrame_pleural_consenso_RMCI derrame_pleural_estad_clínico_radiológico /ORDER=ANALYSIS.
Resources	Processor Time	0:00:00.016
	Elapsed Time	0:00:00.015

```
[DataSet1] C:\Users\Fábio\Desktop\ALEX_SPSS\PLANILHA.sav
```

Statistics

		derrame_pleural_RMCI_1	derrame_pleural_RMCI_2	derrame_pleural_estad_padrão	derrame_pleural_consenso_RMCI	derrame_pleural_estad_clínico_radiológico
N	Valid	34	34	34	34	34
	Missing	0	0	0	0	0

Frequency Table

derrame_pleural_RMCI_1

		Frequency	Percent	Valid Percent	Cumulative Percent
Valid	AUSENTE	31	91,2	91,2	91,2
	PRESENTE	3	8,8	8,8	100,0
	Total	34	100,0	100,0	

derrame_pleural_RMCI_2

		Frequency	Percent	Valid Percent	Cumulative Percent
Valid	AUSENTE	31	91,2	91,2	91,2
	PRESENTE	3	8,8	8,8	100,0
	Total	34	100,0	100,0	

derrame_pleural_estad_padrão

		Frequency	Percent	Valid Percent	Cumulative Percent
Valid	AUSENTE	31	91,2	91,2	91,2
	PRESENTE	3	8,8	8,8	100,0
	Total	34	100,0	100,0	

derrame_pleural_consenso_RMCI

		Frequency	Percent	Valid Percent	Cumulative Percent
Valid	AUSENTE	31	91,2	91,2	91,2
	PRESENTE	3	8,8	8,8	100,0
	Total	34	100,0	100,0	

derrame_pleural_estad_clínico_radiológico

		Frequency	Percent	Valid Percent	Cumulative Percent
Valid	AUSENTE	31	91,2	91,2	91,2
	PRESENTE	3	8,8	8,8	100,0
	Total	34	100,0	100,0	

```
CROSSTABS
  /TABLES=derrame_pleural_estad_clínico_radiológico BY derrame_pleural_con
senso_RMCI
  /FORMAT=AVALUE TABLES
  /STATISTICS=KAPPA
  /CELLS=COUNT
  /COUNT ROUND CELL.
```

Crosstabs

Notes

	Output Created	15-Nov-2016 19h54min40s
	Comments	
Input	Data	C:\Users\Fábio\Desktop\ALEX_ SPSS\PLANILHA.sav
	Active Dataset	DataSet1
	Filter	<none>
	Weight	<none>
	Split File	<none>
	N of Rows in Working Data File	34

Notes

Missing Value Handling	Definition of Missing	User-defined missing values are treated as missing.
	Cases Used	Statistics for each table are based on all the cases with valid data in the specified range(s) for all variables in each table.
	Syntax	CROSSTABS<br>/TABLES=derrame_pleural_estad_clínico_radiológico BY derrame_pleural_consenso_RMCI<br>/FORMAT=AVALUE TABLES<br>/STATISTICS=KAPPA<br>/CELLS=COUNT<br>/COUNT ROUND CELL.
Resources	Processor Time	0:00:00.000
	Elapsed Time	0:00:00.014
	Dimensions Requested	2
	Cells Available	174762

```
[DataSet1] C:\Users\Fábio\Desktop\ALEX_SPSS\PLANILHA.sav
```

Case Processing Summary

	Cases					
	Valid		Missing		Total	
	N	Percent	N	Percent	N	Percent
derrame_pleural_estad_clínico_radiológico * derrame_pleural_consenso_RMCI	34	100,0%	0	,0%	34	100,0%

derrame_pleural_estad_clínico_radiológico * derrame_pleural_consenso_RMCI Crosstabulation

Count

		derrame_pleural_consenso_RMCI		
		AUSENTE	PRESENTE	Total
derrame_pleural_estad_clínico_radiológico	AUSENTE	31	0	31
	PRESENTE	0	3	3
	Total	31	3	34

Symmetric Measures

		Value	Asymp. Std. Error[a]	Approx. T[b]	Approx. Sig.
Measure of Agreement	Kappa	1,000	,000	5,831	,000
	N of Valid Cases	34			

a. Not assuming the null hypothesis.

b. Using the asymptotic standard error assuming the null hypothesis.
