# Supplementary material for: Evaluation of whole-body MRI with diffusion-weighted sequences in the staging of pediatric cancer patients
Source: PLoS One. 2020 Aug 27;15(8):e0238166. doi: 10.1371/journal.pone.0238166 (PMC7451574; doi:10.1371/journal.pone.0238166)
Supplement: S1 File — (ZIP) [file pone.0238166.s002.zip › DADOS_LES╟O_PRIM╡RIA.pdf]

```
FREQUENCIES VARIABLES=LESÃO_PRIMÁRIA_RMCI_1 LESÃO_PRIMÁRIA_RMCI_2 LESÃO_PR
IMÁRIA_ESTAD_PADRÃO LESÃO_PRIMÁRIA_ESTAD_CLÍNICO_RADIOLÓGI
   CO CONSENSO_RMCI_LESÃO_PRIMÁRIA
  /ORDER=ANALYSIS.
```

Frequencies

Notes

	Output Created	15-Nov-2016 16h43min30s
	Comments	
Input	Data	C:\Users\Fábio\Desktop\ALEX_SPSS\PLANILHA.sav
	Active Dataset	DataSet1
	Filter	<none>
	Weight	<none>
	Split File	<none>
	N of Rows in Working Data File	34
Missing Value Handling	Definition of Missing	User-defined missing values are treated as missing.
	Cases Used	Statistics are based on all cases with valid data.
	Syntax	FREQUENCIES VARIABLES=LESÃO_PRIMÁRIA_RMCI_1 LESÃO_PRIMÁRIA_RMCI_2 LESÃO_PRIMÁRIA_ESTAD_PADRÃO LESÃO_PRIMÁRIA_ESTAD_CLÍNICO_RADIOLÓGICO CONSENSO_RMCI_LESÃO_PRIMÁRIA /ORDER=ANALYSIS.
Resources	Processor Time	0:00:00.000
	Elapsed Time	0:00:00.000

```
[DataSet1] C:\Users\Fábio\Desktop\ALEX_SPSS\PLANILHA.sav
```

Statistics

		LESÃO_PRIMÁRIA_RMCI_1	LESÃO_PRIMÁRIA_RMCI_2	LESÃO_PRIMÁRIA_ESTAD_PADRÃO	LESÃO_PRIMÁRIA_ESTAD_CLÍNICO_RADIOLÓGICO	CONSENSO_RMCI_LESÃO_PRIMÁRIA
N	Valid	34	34	34	34	34
	Missing	0	0	0	0	0

Frequency Table

LESÃO_PRIMÁRIA_RMCI_1

		Frequency	Percent	Valid Percent	Cumulative Percent
Valid	AUSENTE	2	5,9	5,9	5,9
	PRESENTE	19	55,9	55,9	61,8
	NÃO APLICÁVEL	13	38,2	38,2	100,0
	Total	34	100,0	100,0	

LESÃO_PRIMÁRIA_RMCI_2

		Frequency	Percent	Valid Percent	Cumulative Percent
Valid	AUSENTE	3	8,8	8,8	8,8
	PRESENTE	18	52,9	52,9	61,8
	NÃO APLICÁVEL	13	38,2	38,2	100,0
	Total	34	100,0	100,0	

LESÃO_PRIMÁRIA_ESTAD_PADRÃO

		Frequency	Percent	Valid Percent	Cumulative Percent
Valid	AUSENTE	2	5,9	5,9	5,9
	PRESENTE	19	55,9	55,9	61,8
	NÃO APLICÁVEL	13	38,2	38,2	100,0
	Total	34	100,0	100,0	

LESÃO_PRIMÁRIA_ESTAD_CLÍNICO_RADIOLÓGICO

		Frequency	Percent	Valid Percent	Cumulative Percent
Valid	AUSENTE	2	5,9	5,9	5,9
	PRESENTE	19	55,9	55,9	61,8
	NÃO APLICÁVEL	13	38,2	38,2	100,0
	Total	34	100,0	100,0	

CONSENSO_RMCI_LESÃO_PRIMÁRIA

		Frequency	Percent	Valid Percent	Cumulative Percent
Valid	NENHUMA METÁSTASE	2	5,9	5,9	5,9
	1 METÁSTASE	19	55,9	55,9	61,8
	100,00	13	38,2	38,2	100,0
	Total	34	100,0	100,0	

```
CROSSTABS
  /TABLES=LESÃO_PRIMÁRIA_ESTAD_CLÍNICO_RADIOLÓGICO BY CONSENSO_RMCI_LESÃO_
PRIMÁRIA
  /FORMAT=AVALUE TABLES
  /STATISTICS=KAPPA
  /CELLS=COUNT TOTAL
  /COUNT ROUND CELL.
```

Crosstabs

Notes

	Output Created	15-Nov-2016 16h46min26s
	Comments	
Input	Data	C:\Users\Fábio\Desktop\ALEX_ SPSS\PLANILHA.sav
	Active Dataset	DataSet1
	Filter	<none>
	Weight	<none>
	Split File	<none>
	N of Rows in Working Data File	34
Missing Value Handling	Definition of Missing	User-defined missing values are treated as missing.
	Cases Used	Statistics for each table are based on all the cases with valid data in the specified range(s) for all variables in each table.
	Syntax	CROSSTABS /TABLES=LESÃO_PRIMÁRIA_ ESTAD_CLÍNICO_RADIOLÓGICO BY CONSENSO_RMCI_LESÃO_ PRIMÁRIA /FORMAT=AVALUE TABLES /STATISTICS=KAPPA /CELLS=COUNT TOTAL /COUNT ROUND CELL.
Resources	Processor Time	0:00:00.031
	Elapsed Time	0:00:00.031
	Dimensions Requested	2
	Cells Available	174762

```
[DataSet1] C:\Users\Fábio\Desktop\ALEX_SPSS\PLANILHA.sav
```

Case Processing Summary

	Cases					
	Valid		Missing		Total	
	N	Percent	N	Percent	N	Percent
LESÃO_PRIMÁRIA_ ESTAD_CLÍNICO_ RADIOLÓGICO * CONSENSO_RMCI_ LESÃO_PRIMÁRIA	34	100,0%	0	,0%	34	100,0%

LESÃO_PRIMÁRIA_ESTAD_CLÍNICO_RADIOLÓGICO * CONSENSO_RMCI_LESÃO_PRIMÁRIA Crosstabulation

			CONSENSO_RMCI_LESÃO_ PRIMÁRIA	
			NENHUMA METÁSTASE	1 METÁSTASE
LESÃO_PRIMÁRIA_ ESTAD_CLÍNICO_ RADIOLÓGICO	AUSENTE	Count	2	0
		% of Total	5,9%	,0%
	PRESENTE	Count	0	19

LESÃO_PRIMÁRIA_ESTAD_CLÍNICO_RADIOLÓGICO * CONSENSO_RMCI_LESÃO_PRIMÁRIA Crosstabulation

			CONSENSO_ RMCI_ LESÃO_ PRIMÁRIA	
			100,00	Total
LESÃO_PRIMÁRIA_ ESTAD_CLÍNICO_ RADIOLÓGICO	AUSENTE	Count	0	2
		% of Total	,0%	5,9%
	PRESENTE	Count	0	19

LESÃO_PRIMÁRIA_ESTAD_CLÍNICO_RADIOLÓGICO * CONSENSO_RMCI_LESÃO_PRIMÁRIA Crosstabulation

			CONSENSO_RMCI_LESÃO_ PRIMÁRIA	
			NENHUMA METÁSTASE	1 METÁSTASE
LESÃO_PRIMÁRIA_ ESTAD_CLÍNICO_ RADIOLÓGICO	PRESENTE	% of Total	,0%	55,9%
	NÃO APLICÁVEL	Count	0	0
		% of Total	,0%	,0%
	Total	Count	2	19
		% of Total	5,9%	55,9%

LESÃO_PRIMÁRIA_ESTAD_CLÍNICO_RADIOLÓGICO * CONSENSO_RMCI_LESÃO_PRIMÁRIA Crosstabulation

			CONSENSO_ RMCI_ LESÃO_ PRIMÁRIA	
			100,00	Total
LESÃO_PRIMÁRIA_ ESTAD_CLÍNICO_ RADIOLÓGICO	PRESENTE	% of Total	,0%	55,9%
	NÃO APLICÁVEL	Count	13	13
		% of Total	38,2%	38,2%
	Total	Count	13	34
		% of Total	38,2%	100,0%

Symmetric Measures

		Value	Asymp. Std. Error[a]	Approx. T[b]	Approx. Sig.
Measure of Agreement	Kappa	1,000	,000	6,781	,000
	N of Valid Cases	34			

a. Not assuming the null hypothesis.

b. Using the asymptotic standard error assuming the null hypothesis.

```
CROSSTABS
  /TABLES=LESÃO_PRIMÁRIA_RMCI_1 BY LESÃO_PRIMÁRIA_RMCI_2
  /FORMAT=AVALUE TABLES
  /STATISTICS=KAPPA
  /CELLS=COUNT TOTAL
  /COUNT ROUND CELL.
```

Crosstabs

Notes

	Output Created	15-Nov-2016 16h47min24s
	Comments	
Input	Data	C:\Users\Fábio\Desktop\ALEX_SPSS\PLANILHA.sav
	Active Dataset	DataSet1
	Filter	<none>
	Weight	<none>
	Split File	<none>
	N of Rows in Working Data File	34
Missing Value Handling	Definition of Missing	User-defined missing values are treated as missing.
	Cases Used	Statistics for each table are based on all the cases with valid data in the specified range(s) for all variables in each table.
	Syntax	CROSSTABS /TABLES=LESÃO_PRIMÁRIA_RMCI_1 BY LESÃO_PRIMÁRIA_RMCI_2 /FORMAT=AVALUE TABLES /STATISTICS=KAPPA /CELLS=COUNT TOTAL /COUNT ROUND CELL.
Resources	Processor Time	0:00:00.031
	Elapsed Time	0:00:00.031
	Dimensions Requested	2
	Cells Available	174762

```
[DataSet1] C:\Users\Fábio\Desktop\ALEX_SPSS\PLANILHA.sav
```

Case Processing Summary

	Cases					
	Valid		Missing		Total	
	N	Percent	N	Percent	N	Percent
LESÃO_PRIMÁRIA_RMCI_1 * LESÃO_PRIMÁRIA_RMCI_2	34	100,0%	0	,0%	34	100,0%

LESÃO_PRIMÁRIA_RMCI_1 * LESÃO_PRIMÁRIA_RMCI_2 Crosstabulation

			LESÃO_PRIMÁRIA_RMCI_2	
			AUSENTE	PRESENTE
LESÃO_PRIMÁRIA_RMCI_1	AUSENTE	Count	2	0
		% of Total	5,9%	,0%
	PRESENTE	Count	1	18
		% of Total	2,9%	52,9%
	NÃO APLICÁVEL	Count	0	0
		% of Total	,0%	,0%

LESÃO_PRIMÁRIA_RMCI_1 * LESÃO_PRIMÁRIA_RMCI_2 Crosstabulation

			LESÃO_PRIMÁRIA_RMCI_2	
			NÃO APLICÁVEL	Total
LESÃO_PRIMÁRIA_RMCI_1	AUSENTE	Count	0	2
		% of Total	,0%	5,9%
	PRESENTE	Count	0	19
		% of Total	,0%	55,9%
	NÃO APLICÁVEL	Count	13	13
		% of Total	38,2%	38,2%

LESÃO_PRIMÁRIA_RMCI_1 * LESÃO_PRIMÁRIA_RMCI_2 Crosstabulation

		LESÃO_PRIMÁRIA_RMCI_2	
		AUSENTE	PRESENTE
Total	Count	3	18
	% of Total	8,8%	52,9%

LESÃO_PRIMÁRIA_RMCI_1 * LESÃO_PRIMÁRIA_RMCI_2 Crosstabulation

		LESÃO_PRIMÁRIA_RMCI_2	
		NÃO APLICÁVEL	Total
Total	Count	13	34
	% of Total	38,2%	100,0%

Symmetric Measures

		Value	Asymp. Std. Error[a]	Approx. T[b]	Approx. Sig.
Measure of Agreement	Kappa	,947	,052	6,616	,000
	N of Valid Cases	34			

a. Not assuming the null hypothesis.

b. Using the asymptotic standard error assuming the null hypothesis.

```
CROSSTABS
  /TABLES=LESÃO_PRIMÁRIA_ESTAD_PADRÃO BY CONSENSO_RMCI_LESÃO_PRIMÁRIA
  /FORMAT=AVALUE TABLES
  /STATISTICS=KAPPA
  /CELLS=COUNT TOTAL
  /COUNT ROUND CELL.
```

Crosstabs

Notes

	Output Created	15-Nov-2016 16h48min31s
	Comments	
Input	Data	C:\Users\Fábio\Desktop\ALEX_ SPSS\PLANILHA.sav
	Active Dataset	DataSet1
	Filter	<none>
	Weight	<none>
	Split File	<none>
	N of Rows in Working Data File	34
Missing Value Handling	Definition of Missing	User-defined missing values are treated as missing.
	Cases Used	Statistics for each table are based on all the cases with valid data in the specified range(s) for all variables in each table.
	Syntax	CROSSTABS /TABLES=LESÃO_PRIMÁRIA_ ESTAD_PADRÃO BY CONSENSO_RMCI_LESÃO_ PRIMÁRIA /FORMAT=AVALUE TABLES /STATISTICS=KAPPA /CELLS=COUNT TOTAL /COUNT ROUND CELL.
Resources	Processor Time	0:00:00.015
	Elapsed Time	0:00:00.015
	Dimensions Requested	2
	Cells Available	174762

```
[DataSet1] C:\Users\Fábio\Desktop\ALEX_SPSS\PLANILHA.sav
```

Case Processing Summary

	Cases					
	Valid		Missing		Total	
	N	Percent	N	Percent	N	Percent
LESÃO_PRIMÁRIA_ ESTAD_PADRÃO * CONSENSO_RMCI_ LESÃO_PRIMÁRIA	34	100,0%	0	,0%	34	100,0%

LESÃO_PRIMÁRIA_ESTAD_PADRÃO * CONSENSO_RMCI_LESÃO_PRIMÁRIA Crosstabulation

			CONSENSO_RMCI_LESÃO_PRIMÁRIA	
			NENHUMA METÁSTASE	1 METÁSTASE
LESÃO_PRIMÁRIA_ESTAD_PADRÃO	AUSENTE	Count	2	0
		% of Total	5,9%	,0%
	PRESENTE	Count	0	19
		% of Total	,0%	55,9%
	NÃO APLICÁVEL	Count	0	0
		% of Total	,0%	,0%
	Total	Count	2	19
		% of Total	5,9%	55,9%

LESÃO_PRIMÁRIA_ESTAD_PADRÃO * CONSENSO_RMCI_LESÃO_PRIMÁRIA Crosstabulation

			CONSENSO_RMCI_LESÃO_PRIMÁRIA	
			100,00	Total
LESÃO_PRIMÁRIA_ESTAD_PADRÃO	AUSENTE	Count	0	2
		% of Total	,0%	5,9%
	PRESENTE	Count	0	19
		% of Total	,0%	55,9%
	NÃO APLICÁVEL	Count	13	13
		% of Total	38,2%	38,2%
	Total	Count	13	34
		% of Total	38,2%	100,0%

Symmetric Measures

		Value	Asymp. Std. Error[a]	Approx. T[b]	Approx. Sig.
Measure of Agreement	Kappa	1,000	,000	6,781	,000
	N of Valid Cases	34			

a. Not assuming the null hypothesis.

b. Using the asymptotic standard error assuming the null hypothesis.
