# Supplementary material for: Evaluation of whole-body MRI with diffusion-weighted sequences in the staging of pediatric cancer patients
Source: PLoS One. 2020 Aug 27;15(8):e0238166. doi: 10.1371/journal.pone.0238166 (PMC7451574; doi:10.1371/journal.pone.0238166)
Supplement: S1 File — (ZIP) [file pone.0238166.s002.zip › DADOS_META_LINFONODAL - Copia.pdf]

```
FREQUENCIES VARIABLES=META_LINFONODAL_RMCI_1 META_LINFONODAL_RMCI_2 META_L
INFONODAL_ESTAD_PADRÃO META_LINFONODAL_ESTAD_CLÍNICO_RADIO
   LÓGICO META_LINF_CONSENSO
  /ORDER=ANALYSIS.
```

Frequencies

Notes

	Output Created	15-Nov-2016 20h5min15s
	Comments	
Input	Data	C:\Users\Fábio\Desktop\ALEX_SPSS\PLANILHA.sav
	Active Dataset	DataSet1
	Filter	<none>
	Weight	<none>
	Split File	<none>
	N of Rows in Working Data File	34
Missing Value Handling	Definition of Missing	User-defined missing values are treated as missing.
	Cases Used	Statistics are based on all cases with valid data.
	Syntax	FREQUENCIES VARIABLES=META_LINFONODAL_RMCI_1 META_LINFONODAL_RMCI_2 META_LINFONODAL_ESTAD_PADRÃO META_LINFONODAL_ESTAD_CLÍNICO_RADIOLÓGICO META_LINF_CONSENSO /ORDER=ANALYSIS.
Resources	Processor Time	0:00:00.000
	Elapsed Time	0:00:00.000

```
[DataSet1] C:\Users\Fábio\Desktop\ALEX_SPSS\PLANILHA.sav
```

Statistics

		META_LINFONODAL RMCI_1	META_LINFONODAL RMCI_2	META_LINFONODAL_ESTAD_PADRÃO	META_LINFONODAL_ESTAD_CLÍNICO_RADIOLÓGICO	META_LINF_CONSENSO
N	Valid	34	34	34	34	34
	Missing	0	0	0	0	0

Frequency Table

META_LINFONODAL_RMCI_1

		Frequency	Percent	Valid Percent	Cumulative Percent
Valid	NENHUMA CADEIA	26	76,5	76,5	76,5
	1 CADEIA	1	2,9	2,9	79,4
	2 OU MAIS CADEIAS	1	2,9	2,9	82,4
	NÃO APLICÁVEL	6	17,6	17,6	100,0
	Total	34	100,0	100,0	

META_LINFONODAL_RMCI_2

		Frequency	Percent	Valid Percent	Cumulative Percent
Valid	NENHUMA CADEIA	27	79,4	79,4	79,4
	2 OU MAIS CADEIAS	1	2,9	2,9	82,4
	NÃO APLICÁVEL	6	17,6	17,6	100,0
	Total	34	100,0	100,0	

META_LINFONODAL_ESTAD_PADRÃO

		Frequency	Percent	Valid Percent	Cumulative Percent
Valid	NENHUMA CADEIA	24	70,6	70,6	70,6
	1 CADEIA	2	5,9	5,9	76,5
	2 OU MAIS CADEIAS	2	5,9	5,9	82,4
	NÃO APLICÁVEL	6	17,6	17,6	100,0
	Total	34	100,0	100,0	

META_LINFONODAL_ESTAD_CLÍNICO_RADIOLÓGICO

		Frequency	Percent	Valid Percent	Cumulative Percent
Valid	NENHUMA CADEIA	25	73,5	73,5	73,5
	1 CADEIA	1	2,9	2,9	76,5
	2 OU MAIS CADEIAS	2	5,9	5,9	82,4
	NÃO APLICÁVEL	6	17,6	17,6	100,0
	Total	34	100,0	100,0	

META_LINF_CONSENSO

		Frequency	Percent	Valid Percent	Cumulative Percent
Valid	NENHUMA CADEIA	26	76,5	76,5	76,5
	1 CADEIA	1	2,9	2,9	79,4
	2 OU MAIS CADEIAS	1	2,9	2,9	82,4
	NÃO APLICÁVEL	6	17,6	17,6	100,0
	Total	34	100,0	100,0	

```
CROSSTABS
  /TABLES=META_LINF_CONSENSO BY META_LINFONODAL_ESTAD_CLÍNICO_RADIOLÓGICO
  /FORMAT=AVALUE TABLES
  /STATISTICS=KAPPA
  /CELLS=COUNT TOTAL
```

```
/COUNT ROUND CELL.
```

Crosstabs

Notes

	Output Created	15-Nov-2016 20h5min35s
	Comments	
Input	Data	C:\Users\Fábio\Desktop\ALEX_ SPSS\PLANILHA.sav
	Active Dataset	DataSet1
	Filter	<none>
	Weight	<none>
	Split File	<none>
	N of Rows in Working Data File	34
Missing Value Handling	Definition of Missing	User-defined missing values are treated as missing.
	Cases Used	Statistics for each table are based on all the cases with valid data in the specified range(s) for all variables in each table.
	Syntax	CROSSTABS<br>/TABLES=META_LINF_CONSENSO BY META_LINFONODAL_ESTAD_ CLÍNICO_RADIOLÓGICO<br>/FORMAT=AVALUE TABLES<br>/STATISTICS=KAPPA<br>/CELLS=COUNT TOTAL<br>/COUNT ROUND CELL.
Resources	Processor Time	0:00:00.000
	Elapsed Time	0:00:00.000
	Dimensions Requested	2
	Cells Available	174762

```
[DataSet1] C:\Users\Fábio\Desktop\ALEX_SPSS\PLANILHA.sav
```

Case Processing Summary

	Cases					
	Valid		Missing		Total	
	N	Percent	N	Percent	N	Percent
META_LINF_CONSENSO * META_LINFONODAL_ ESTAD_CLÍNICO_ RADIOLÓGICO	34	100,0%	0	,0%	34	100,0%

META_LINF_CONSENSO * META_LINFONODAL_ESTAD_CLÍNICO_RADIOLÓGICO Crosstabulation

			META_LINFONODAL_ESTAD_CLÍNICO_RADIOLÓGICO	
			NENHUMA CADEIA	1 CADEIA
META_LINF_CONSENSO	NENHUMA CADEIA	Count	25	0
		% of Total	73,5%	,0%
	1 CADEIA	Count	0	1
		% of Total	,0%	2,9%

META_LINF_CONSENSO * META_LINFONODAL_ESTAD_CLÍNICO_RADIOLÓGICO Crosstabulation

			META_LINFONODAL_ESTAD_CLÍNICO_RADIOLÓGICO	
			2 OU MAIS CADEIAS	NÃO APLICÁVEL
META_LINF_CONSENSO	NENHUMA CADEIA	Count	1	0
		% of Total	2,9%	,0%
	1 CADEIA	Count	0	0
		% of Total	,0%	,0%

META_LINF_CONSENSO * META_LINFONODAL_ESTAD_CLÍNICO_RADIOLÓGICO Crosstabulation

			Total
META_LINF_CONSENSO	NENHUMA CADEIA	Count	26
		% of Total	76,5%
	1 CADEIA	Count	1
		% of Total	2,9%

META_LINF_CONSENSO * META_LINFONODAL_ESTAD_CLÍNICO_RADIOLÓGICO Crosstabulation

			META_LINFONODAL_ESTAD_CLÍNICO_RADIOLÓGICO	
			NENHUMA CADEIA	1 CADEIA
META_LINF_CONSENSO	2 OU MAIS CADEIAS	Count	0	0
		% of Total	,0%	,0%
	NÃO APLICÁVEL	Count	0	0
		% of Total	,0%	,0%
	Total	Count	25	1
		% of Total	73,5%	2,9%

META_LINF_CONSENSO * META_LINFONODAL_ESTAD_CLÍNICO_RADIOLÓGICO Crosstabulation

			META_LINFONODAL_ESTAD_CLÍNICO_RADIOLÓGICO	
			2 OU MAIS CADEIAS	NÃO APLICÁVEL
META_LINF_CONSENSO	2 OU MAIS CADEIAS	Count	1	0
		% of Total	2,9%	,0%
	NÃO APLICÁVEL	Count	0	6
		% of Total	,0%	17,6%
	Total	Count	2	6
		% of Total	5,9%	17,6%

META_LINF_CONSENSO * META_LINFONODAL_ESTAD_CLÍNICO_RADIOLÓGICO Crosstabulation

			Total
META_LINF_CONSENSO	2 OU MAIS CADEIAS	Count	1
		% of Total	2,9%
	NÃO APLICÁVEL	Count	6
		% of Total	17,6%
	Total	Count	34
		% of Total	100,0%

Symmetric Measures

		Value	Asymp. Std. Error[a]	Approx. T[b]	Approx. Sig.
Measure of Agreement	Kappa	,927	,071	7,026	,000
	N of Valid Cases	34			

a. Not assuming the null hypothesis.

b. Using the asymptotic standard error assuming the null hypothesis.
