# Supplementary material for: Evaluation of whole-body MRI with diffusion-weighted sequences in the staging of pediatric cancer patients
Source: PLoS One. 2020 Aug 27;15(8):e0238166. doi: 10.1371/journal.pone.0238166 (PMC7451574; doi:10.1371/journal.pone.0238166)
Supplement: S1 File — (ZIP) [file pone.0238166.s002.zip › DADOS_META_OSSEA_REVER - Copia.pdf]

```
FREQUENCIES VARIABLES=META_ÓSSEA_RMC1 META_ÓSSEA_RMCI_2 META_ÓSSEA_ESTAD_P
ADRÃO META_ÓSSEA_CONSENSO_RMCI META_ÓSSEA_ESTAD_CLÍNICO_RA
   DIOLÓGICO
  /ORDER=ANALYSIS.
```

Frequencies

Notes

	Output Created	15-Nov-2016 20h8min14s
	Comments	
Input	Data	C:\Users\Fábio\Desktop\ALEX_SPSS\PLANILHA.sav
	Active Dataset	DataSet1
	Filter	<none>
	Weight	<none>
	Split File	<none>
	N of Rows in Working Data File	34
Missing Value Handling	Definition of Missing	User-defined missing values are treated as missing.
	Cases Used	Statistics are based on all cases with valid data.
	Syntax	FREQUENCIES VARIABLES=META_ÓSSEA_RMC1 META_ÓSSEA_RMCI_2 META_ÓSSEA_ESTAD_PADRÃO META_ÓSSEA_CONSENSO_RMCI META_ÓSSEA_ESTAD_CLÍNICO_RADIOLÓGICO /ORDER=ANALYSIS.
Resources	Processor Time	0:00:00.000
	Elapsed Time	0:00:00.000

```
[DataSet1] C:\Users\Fábio\Desktop\ALEX_SPSS\PLANILHA.sav
```

Statistics

		META_ÓSSEA_RMC1	META_ÓSSEA_RMCI_2	META_ÓSSEA_ESTAD_PADRÃO	META_ÓSSEA_CONSENSO_RMCI	META_ÓSSEA_ESTAD_CLÍNICO_RADIOLÓGICO
N	Valid	34	34	34	34	34
	Missing	0	0	0	0	0

Frequency Table

META_ÓSSEA_RMC1

		Frequency	Percent	Valid Percent	Cumulative Percent
Valid	NENHUMA METÁSTASE	29	85,3	85,3	85,3
	1 METÁSTASE	3	8,8	8,8	94,1
	2 METÁSTASES	1	2,9	2,9	97,1

META_ÓSSEA_RMC1

		Frequency	Percent	Valid Percent	Cumulative Percent
Valid	100,00	1	2,9	2,9	100,0
	Total	34	100,0	100,0	

META_ÓSSEA_RMCI_2

		Frequency	Percent	Valid Percent	Cumulative Percent
Valid	NENHUMA METÁSTASE	26	76,5	76,5	76,5
	1 METÁSTASE	3	8,8	8,8	85,3
	2 METÁSTASES	1	2,9	2,9	88,2
	3 OU MAIS METÁSTASES	3	8,8	8,8	97,1
	100,00	1	2,9	2,9	100,0
	Total	34	100,0	100,0	

META_ÓSSEA_ESTAD_PADRÃO

		Frequency	Percent	Valid Percent	Cumulative Percent
Valid	NENHUMA METÁSTASE	28	82,4	82,4	82,4
	2 METÁSTASES	3	8,8	8,8	91,2
	3 OU MAIS METÁSTASES	2	5,9	5,9	97,1
	100,00	1	2,9	2,9	100,0
	Total	34	100,0	100,0	

META_ÓSSEA_CONSENSO_RMCI

		Frequency	Percent	Valid Percent	Cumulative Percent
Valid	NENHUMA METÁSTASE	26	76,5	76,5	76,5
	1 METÁSTASE	3	8,8	8,8	85,3
	2 METÁSTASES	1	2,9	2,9	88,2
	3 OU MAIS METÁSTASES	3	8,8	8,8	97,1
	100,00	1	2,9	2,9	100,0
	Total	34	100,0	100,0	

META_ÓSSEA_ESTAD_CLÍNICO_RADIOLÓGICO

		Frequency	Percent	Valid Percent	Cumulative Percent
Valid	NENHUMA METÁSTASE	30	88,2	88,2	88,2
	1 METÁSTASE	1	2,9	2,9	91,2
	3 OU MAIS METÁSTASES	2	5,9	5,9	97,1
	100,00	1	2,9	2,9	100,0
	Total	34	100,0	100,0	

```
CROSSTABS
  /TABLES=META_ÓSSEA_CONSENSO_RMCI BY META_ÓSSEA_ESTAD_CLÍNICO_RADIOLÓGICO
  /FORMAT=AVALUE TABLES
  /STATISTICS=KAPPA
  /CELLS=COUNT TOTAL
```

```
/COUNT ROUND CELL.
```

Crosstabs

Notes

	Output Created	15-Nov-2016 20h8min38s
	Comments	
Input	Data	C:\Users\Fábio\Desktop\ALEX_ SPSS\PLANILHA.sav
	Active Dataset	DataSet1
	Filter	<none>
	Weight	<none>
	Split File	<none>
	N of Rows in Working Data File	34
Missing Value Handling	Definition of Missing	User-defined missing values are treated as missing.
	Cases Used	Statistics for each table are based on all the cases with valid data in the specified range(s) for all variables in each table.
	Syntax	CROSSTABS<br>/TABLES=META_ÓSSEA_ CONSENSO_RMCI BY META_ÓSSEA_ESTAD_CLÍNICO_ RADIOLÓGICO<br>/FORMAT=AVALUE TABLES<br>/STATISTICS=KAPPA<br>/CELLS=COUNT TOTAL<br>/COUNT ROUND CELL.
Resources	Processor Time	0:00:00.016
	Elapsed Time	0:00:00.017
	Dimensions Requested	2
	Cells Available	174762

```
[DataSet1] C:\Users\Fábio\Desktop\ALEX_SPSS\PLANILHA.sav
```

Case Processing Summary

	Cases					
	Valid		Missing		Total	
	N	Percent	N	Percent	N	Percent
META_ÓSSEA_ CONSENSO_RMCI * META_ÓSSEA_ESTAD_ CLÍNICO_RADIOLÓGICO	34	100,0%	0	,0%	34	100,0%

META_ÓSSEA_CONSENSO_RMCI * META_ÓSSEA_ESTAD_CLÍNICO_RADIOLÓGICO Crosstabulation

			META_ÓSSEA_ESTAD_ CLÍNICO_RADIOLÓGICO	
			NENHUMA METÁSTASE	1 METÁSTASE
META_ÓSSEA_ CONSENSO_RMCI	NENHUMA METÁSTASE	Count	26	0
		% of Total	76,5%	,0%
	1 METÁSTASE	Count	2	1
		% of Total	5,9%	2,9%

META_ÓSSEA_CONSENSO_RMCI * META_ÓSSEA_ESTAD_CLÍNICO_RADIOLÓGICO Crosstabulation

			META_ÓSSEA_ESTAD_ CLÍNICO_RADIOLÓGICO	
			3 OU MAIS METÁSTASES	100,00
META_ÓSSEA_ CONSENSO_RMCI	NENHUMA METÁSTASE	Count	0	0
		% of Total	,0%	,0%
	1 METÁSTASE	Count	0	0
		% of Total	,0%	,0%

META_ÓSSEA_CONSENSO_RMCI * META_ÓSSEA_ESTAD_CLÍNICO_RADIOLÓGICO Crosstabulation

			Total
META_ÓSSEA_ CONSENSO_RMCI	NENHUMA METÁSTASE	Count	26
		% of Total	76,5%
	1 METÁSTASE	Count	3
		% of Total	8,8%

META_ÓSSEA_CONSENSO_RMCI * META_ÓSSEA_ESTAD_CLÍNICO_RADIOLÓGICO Crosstabulation

			META_ÓSSEA_ESTAD_ CLÍNICO_RADIOLÓGICO	
			NENHUMA METÁSTASE	1 METÁSTASE
META_ÓSSEA_ CONSENSO_RMCI	2 METÁSTASES	Count	1	0
		% of Total	2,9%	,0%
	3 OU MAIS METÁSTASES	Count	1	0
		% of Total	2,9%	,0%
	100,00	Count	0	0
		% of Total	,0%	,0%
	Total	Count	30	1
		% of Total	88,2%	2,9%

META_ÓSSEA_CONSENSO_RMCI * META_ÓSSEA_ESTAD_CLÍNICO_RADIOLÓGICO Crosstabulation

			META_ÓSSEA_ESTAD_ CLÍNICO_RADIOLÓGICO	
			3 OU MAIS METÁSTASES	100,00
META_ÓSSEA_ CONSENSO_RMCI	2 METÁSTASES	Count	0	0
		% of Total	,0%	,0%
	3 OU MAIS METÁSTASES	Count	2	0
		% of Total	5,9%	,0%
	100,00	Count	0	1
		% of Total	,0%	2,9%
	Total	Count	2	1
		% of Total	5,9%	2,9%

META_ÓSSEA_CONSENSO_RMCI * META_ÓSSEA_ESTAD_CLÍNICO_RADIOLÓGICO Crosstabulation

			Total
META_ÓSSEA_ CONSENSO_RMCI	2 METÁSTASES	Count	1
		% of Total	2,9%
	3 OU MAIS METÁSTASES	Count	3
		% of Total	8,8%
	100,00	Count	1
		% of Total	2,9%
	Total	Count	34
		% of Total	100,0%

Symmetric Measures

		Value
Measure of Agreement	Kappa	.[a]
	N of Valid Cases	34

a. Kappa statistics cannot be computed.They require a symmetric 2-way table in which the values of the first variable match the values of the second variable.
