# Supplementary material for: Evaluation of whole-body MRI with diffusion-weighted sequences in the staging of pediatric cancer patients
Source: PLoS One. 2020 Aug 27;15(8):e0238166. doi: 10.1371/journal.pone.0238166 (PMC7451574; doi:10.1371/journal.pone.0238166)
Supplement: S1 File — (ZIP) [file pone.0238166.s002.zip › DADOS_PULM╟O_REVER - Copia.pdf]

```
FREQUENCIES VARIABLES=META_PULMÃO_RMC1 META_PULMÃO_RMCI_2 META_PULMÃO_ESTA
D_PADRÃO META_PULMÃO_ESTAD_CLÍNICO_RADIOLÓGICO META_PULMÃO
   _CONSENSO
  /ORDER=ANALYSIS.
```

Frequencies

Notes

	Output Created	15-Nov-2016 20h6min58s
	Comments	
Input	Data	C:\Users\Fábio\Desktop\ALEX_ SPSS\PLANILHA.sav
	Active Dataset	DataSet1
	Filter	<none>
	Weight	<none>
	Split File	<none>
	N of Rows in Working Data File	34
Missing Value Handling	Definition of Missing	User-defined missing values are treated as missing.
	Cases Used	Statistics are based on all cases with valid data.
	Syntax	FREQUENCIES VARIABLES=META_PULMÃO_ RMC1 META_PULMÃO_RMCI_2 META_PULMÃO_ESTAD_PADRÃO META_PULMÃO_ESTAD_CLÍNICO_ RADIOLÓGICO META_PULMÃO_CONSENSO /ORDER=ANALYSIS.
Resources	Processor Time	0:00:00.015
	Elapsed Time	0:00:00.018

```
[DataSet1] C:\Users\Fábio\Desktop\ALEX_SPSS\PLANILHA.sav
```

Statistics

		META_ PULMÃO_ RMC1	META_ PULMÃO_ RMCI_2	META_ PULMÃO_ ESTAD_ PADRÃO	META_ PULMÃO_ ESTAD_ CLÍNICO_ RADIOLÓGIC O	META_ PULMÃO_ CONSENSO
N	Valid	34	34	34	34	34
	Missing	0	0	0	0	0

Frequency Table

META_PULMÃO_RMC1

		Frequency	Percent	Valid Percent	Cumulative Percent
Valid	NENHUMA METÁSTASE	33	97,1	97,1	97,1
	1 METÁSTASE	1	2,9	2,9	100,0
	Total	34	100,0	100,0	

META_PULMÃO_RMCI_2

		Frequency	Percent	Valid Percent	Cumulative Percent
Valid	NENHUMA METÁSTASE	33	97,1	97,1	97,1
	1 METÁSTASE	1	2,9	2,9	100,0
	Total	34	100,0	100,0	

META_PULMÃO_ESTAD_PADRÃO

		Frequency	Percent	Valid Percent	Cumulative Percent
Valid	NENHUMA METÁSTASE	23	67,6	67,6	67,6
	3 OU MAIS METÁSTASES	3	8,8	8,8	76,5
	MICRONÓDULOS INESPECÍFICO	8	23,5	23,5	100,0
	Total	34	100,0	100,0	

META_PULMÃO_ESTAD_CLÍNICO_RADIOLÓGICO

		Frequency	Percent	Valid Percent	Cumulative Percent
Valid	NENHUMA METÁSTASE	31	91,2	91,2	91,2
	3 OU MAIS METÁSTASES	3	8,8	8,8	100,0
	Total	34	100,0	100,0	

META_PULMÃO_CONSENSO

		Frequency	Percent	Valid Percent	Cumulative Percent
Valid	NENHUMA METÁSTASE	33	97,1	97,1	97,1
	1 METÁSTASE	1	2,9	2,9	100,0
	Total	34	100,0	100,0	

```
CROSSTABS
  /TABLES=META_PULMÃO_ESTAD_CLÍNICO_RADIOLÓGICO BY META_PULMÃO_CONSENSO
  /FORMAT=AVALUE TABLES
  /STATISTICS=KAPPA
  /CELLS=COUNT TOTAL
  /COUNT ROUND CELL.
```

Crosstabs

Notes

Output Created	15-Nov-2016 20h7min22s
Comments	

Notes

Input	Data	C:\Users\Fábio\Desktop\ALEX_SPSS\PLANILHA.sav
	Active Dataset	DataSet1
	Filter	<none>
	Weight	<none>
	Split File	<none>
	N of Rows in Working Data File	34
Missing Value Handling	Definition of Missing	User-defined missing values are treated as missing.
	Cases Used	Statistics for each table are based on all the cases with valid data in the specified range(s) for all variables in each table.
	Syntax	CROSSTABS<br>/TABLES=META_PULMÃO_ESTAD_CLÍNICO_RADIOLÓGICO BY META_PULMÃO_CONSENSO<br>/FORMAT=AVALUE TABLES<br>/STATISTICS=KAPPA<br>/CELLS=COUNT TOTAL<br>/COUNT ROUND CELL.
Resources	Processor Time	0:00:00.000
	Elapsed Time	0:00:00.000
	Dimensions Requested	2
	Cells Available	174762

```
[DataSet1] C:\Users\Fábio\Desktop\ALEX_SPSS\PLANILHA.sav
```

Case Processing Summary

	Cases					
	Valid		Missing		Total	
	N	Percent	N	Percent	N	Percent
META_PULMÃO_ESTAD_CLÍNICO_RADIOLÓGICO * META_PULMÃO_CONSENSO	34	100,0%	0	,0%	34	100,0%

META_PULMÃO_ESTAD_CLÍNICO_RADIOLÓGICO * META_PULMÃO_CONSENSO Crosstabulation

			META_PULMÃO_CONSENSO	
			NENHUMA METÁSTASE	1 METÁSTASE
META_PULMÃO_ESTAD_CLÍNICO_RADIOLÓGICO	NENHUMA METÁSTASE	Count	31	0
		% of Total	91,2%	,0%
	3 OU MAIS METÁSTASES	Count	2	1
		% of Total	5,9%	2,9%
Total		Count	33	1
		% of Total	97,1%	2,9%

META_PULMÃO_ESTAD_CLÍNICO_RADIOLÓGICO * META_PULMÃO_CONSENSO Crosstabulation

			Total
META_PULMÃO_ESTAD_CLÍNICO_RADIOLÓGICO	NENHUMA METÁSTASE	Count	31
		% of Total	91,2%
	3 OU MAIS METÁSTASES	Count	3
		% of Total	8,8%
Total		Count	34
		% of Total	100,0%

Symmetric Measures

		Value
Measure of Agreement	Kappa	.[a]
N of Valid Cases		34

a. Kappa statistics cannot be computed.They require a symmetric 2-way table in which the values of the first variable match the values of the second variable.
