# Supplementary material for: Evaluation of whole-body MRI with diffusion-weighted sequences in the staging of pediatric cancer patients
Source: PLoS One. 2020 Aug 27;15(8):e0238166. doi: 10.1371/journal.pone.0238166 (PMC7451574; doi:10.1371/journal.pone.0238166)
Supplement: S1 File — (ZIP) [file pone.0238166.s002.zip › DADOS_RENAL_REVER - Copia.pdf]

```
FREQUENCIES VARIABLES=nódulo_renal_rmci_1 nódulo_renal_rmci_2 nódulo_renal
_estad_padrão nódulo_renal_estad_clínico_radiológico RENAL
   _CONSENSO
  /ORDER=ANALYSIS.
```

# Frequencies

**Notes**

| | | |
|---|---|---|
| | Output Created | 15-Nov-2016 19h57min59s |
| | Comments | |
| Input | Data | C:\Users\Fábio\Desktop\ALEX_SPSS\PLANILHA.sav |
| | Active Dataset | DataSet1 |
| | Filter | <none> |
| | Weight | <none> |
| | Split File | <none> |
| | N of Rows in Working Data File | 34 |
| Missing Value Handling | Definition of Missing | User-defined missing values are treated as missing. |
| | Cases Used | Statistics are based on all cases with valid data. |
| | Syntax | FREQUENCIES VARIABLES=nódulo_renal_rmci_1 nódulo_renal_rmci_2 nódulo_renal_estad_padrão nódulo_renal_estad_clínico_radiológico RENAL_CONSENSO /ORDER=ANALYSIS. |
| Resources | Processor Time | 0:00:00.016 |
| | Elapsed Time | 0:00:00.016 |

```
[DataSet1] C:\Users\Fábio\Desktop\ALEX_SPSS\PLANILHA.sav
```

**Statistics**

| | | nódulo_renal_rmci_1 | nódulo_renal_rmci_2 | nódulo_renal_estad_padrão | nódulo_renal_estad_clínico_radiológico | RENAL_CONSENSO |
|---|---|---|---|---|---|---|
| N | Valid | 34 | 34 | 34 | 34 | 34 |
| | Missing | 0 | 0 | 0 | 0 | 0 |

# Frequency Table

**nódulo_renal_rmci_1**

| | | Frequency | Percent | Valid Percent | Cumulative Percent |
|---|---|---|---|---|---|
| Valid | AUSENTE | 32 | 94,1 | 94,1 | 94,1 |
| | PRESENTE | 2 | 5,9 | 5,9 | 100,0 |
| | Total | 34 | 100,0 | 100,0 | |

**nódulo_renal_rmci_2**

| | | Frequency | Percent | Valid Percent | Cumulative Percent |
|---|---|---|---|---|---|
| Valid | AUSENTE | 33 | 97,1 | 97,1 | 97,1 |
| | PRESENTE | 1 | 2,9 | 2,9 | 100,0 |
| | Total | 34 | 100,0 | 100,0 | |

**nódulo_renal_estad_padrão**

| | | Frequency | Percent | Valid Percent | Cumulative Percent |
|---|---|---|---|---|---|
| Valid | AUSENTE | 34 | 100,0 | 100,0 | 100,0 |

**nódulo_renal_estad_clínico_radiológico**

| | | Frequency | Percent | Valid Percent | Cumulative Percent |
|---|---|---|---|---|---|
| Valid | AUSENTE | 34 | 100,0 | 100,0 | 100,0 |

**RENAL_CONSENSO**

| | | Frequency | Percent | Valid Percent | Cumulative Percent |
|---|---|---|---|---|---|
| Valid | AUSENTE | 32 | 94,1 | 94,1 | 94,1 |
| | PRESENTE | 2 | 5,9 | 5,9 | 100,0 |
| | Total | 34 | 100,0 | 100,0 | |

```
CROSSTABS
  /TABLES=nódulo_renal_estad_clínico_radiológico BY RENAL_CONSENSO
  /FORMAT=AVALUE TABLES
  /STATISTICS=KAPPA
  /CELLS=COUNT TOTAL
  /COUNT ROUND CELL.
```

# Crosstabs

**Notes**

| | | |
|---|---|---|
| | Output Created | 15-Nov-2016 19h58min42s |
| | Comments | |
| Input | Data | C:\Users\Fábio\Desktop\ALEX_ SPSS\PLANILHA.sav |
| | Active Dataset | DataSet1 |
| | Filter | <none> |
| | Weight | <none> |
| | Split File | <none> |
| | N of Rows in Working Data File | 34 |
| Missing Value Handling | Definition of Missing | User-defined missing values are treated as missing. |
| | Cases Used | Statistics for each table are based on all the cases with valid data in the specified range(s) for all variables in each table. |

**Notes**

| | | |
|---|---|---|
| | Syntax | CROSSTABS<br>/TABLES=nódulo_renal_estad_clínico_radiológico BY RENAL_CONSENSO<br>/FORMAT=AVALUE TABLES<br>/STATISTICS=KAPPA<br>/CELLS=COUNT TOTAL<br>/COUNT ROUND CELL. |
| Resources | Processor Time | 0:00:00.000 |
| | Elapsed Time | 0:00:00.000 |
| | Dimensions Requested | 2 |
| | Cells Available | 174762 |

```
[DataSet1] C:\Users\Fábio\Desktop\ALEX_SPSS\PLANILHA.sav
```

**Warnings**

| |
|---|
| No measures of association are computed for the crosstabulation of nódulo_renal_estad_clínico_radiológico * RENAL_CONSENSO. At least one variable in each 2-way table upon which measures of association are computed is a constant. |

**Case Processing Summary**

| | Cases | | | | | |
|---|---|---|---|---|---|---|
| | Valid | | Missing | | Total | |
| | N | Percent | N | Percent | N | Percent |
| nódulo_renal_estad_clínico_radiológico * RENAL_CONSENSO | 34 | 100,0% | 0 | ,0% | 34 | 100,0% |

**nódulo_renal_estad_clínico_radiológico * RENAL_CONSENSO Crosstabulation**

| | | | RENAL_CONSENSO | | |
|---|---|---|---|---|---|
| | | | AUSENTE | PRESENTE | Total |
| nódulo_renal_estad_clínico_radiológico | AUSENTE | Count | 32 | 2 | 34 |
| | | % of Total | 94,1% | 5,9% | 100,0% |
| | Total | Count | 32 | 2 | 34 |
| | | % of Total | 94,1% | 5,9% | 100,0% |

**Symmetric Measures**

| | | Value |
|---|---|---|
| Measure of Agreement | Kappa | .[a] |
| | N of Valid Cases | 34 |

a. No statistics are computed because nódulo_renal_estad_clínico_radiológico is a constant.
